# Models and approaches for building knowledge translation capacity and capability in health services: a scoping review

**DOI:** 10.1186/s13012-024-01336-0

**Published:** 2024-01-29

**Authors:** Olivia King, Emma West, Laura Alston, Hannah Beks, Michele Callisaya, Catherine E. Huggins, Margaret Murray, Kevin Mc Namara, Michael Pang, Warren Payne, Anna Peeters, Mia Pithie, Alesha M. Sayner, Anna Wong Shee

**Affiliations:** 1 Western Alliance, Warrnambool, VIC Australia; 2 Barwon Health, Geelong, VIC Australia; 3 Deakin University, Deakin Rural Health, PO Box 281, Geelong, Warrnambool, VIC Australia; 4 Monash University, Monash Centre for Scholarship in Health Education, Clayton, VIC Australia; 5 Deakin University, Institute for Mental and Physical Health and Clinical Translation, Geelong, VIC Australia; 6 Research Unit, Colac Area Health, Colac, VIC Australia; 7 Peninsula Clinical School, Central Clinical School, Frankston, VIC Australia; 8 National Centre for Healthy Ageing, Melbourne, VIC Australia; 9 Global Centre for Preventive Health and Nutrition, Deakin University, Institute for Health Transformation, Geelong, VIC Australia; 10 Grampians Health, Ballarat, VIC Australia; 11 Deakin University, Institute for Health Transformation, Geelong, VIC Australia

**Keywords:** Capacity building, Knowledge translation, Implementation, Healthcare, Scoping review

## Abstract

**Background:**

Building healthcare service and health professionals’ capacity and capability to rapidly translate research evidence into health practice is critical to the effectiveness and sustainability of healthcare systems. This review scoped the literature describing programmes to build knowledge translation capacity and capability in health professionals and healthcare services, and the evidence supporting these.

**Methods:**

This scoping review was undertaken using the Joanna Briggs Institute scoping review methodology. Four research databases (Ovid MEDLINE, CINAHL, Embase, and PsycInfo) were searched using a pre-determined strategy. Eligible studies described a programme implemented in healthcare settings to build health professional or healthcare service knowledge translation capacity and capability. Abstracts and full texts considered for inclusion were screened by two researchers. Data from included papers were extracted using a bespoke tool informed by the scoping review questions.

**Results:**

Database searches yielded 10,509 unique citations, of which 136 full texts were reviewed. Thirty-four papers were included, with three additional papers identified on citation searching, resulting in 37 papers describing 34 knowledge translation capability building programmes.

Programmes were often multifaceted, comprising a combination of two or more strategies including education, dedicated implementation support roles, strategic research-practice partnerships and collaborations, co-designed knowledge translation capability building programmes, and dedicated funding for knowledge translation. Many programmes utilised experiential and collaborative learning, and targeted either individual, team, organisational, or system levels of impact. Twenty-seven programmes were evaluated formally using one or more data collection methods. Outcomes measured varied significantly and included participant self-reported outcomes, perceived barriers and enablers of knowledge translation, milestone achievement and behaviour change. All papers reported that programme objectives were achieved to varying degrees.

**Conclusions:**

Knowledge translation capacity and capability building programmes in healthcare settings are multifaceted, often include education to facilitate experiential and collaborative learning, and target individual, team, organisational, or supra-organisational levels of impact. Although measured differently across the programmes, the outcomes were positive. The sustainability of programmes and outcomes may be undermined by the lack of long-term funding and inconsistent evaluation. Future research is required to develop evidence-informed frameworks to guide methods and outcome measures for short-, medium- and longer-term programme evaluation at the different structural levels.

**Supplementary Information:**

The online version contains supplementary material available at 10.1186/s13012-024-01336-0.

Contributions to the literature

- Programmes to build the capacity and capability of healthcare services and health professionals to translate research rapidly and effectively into practice are critical. This review identified 34 unique programmes.
- The programmes applied a range of multifaceted strategies that generally targeted one level of impact (individual, team, organisational, and supra-organisational).
- The types of outcomes measured varied significantly and most produced positive changes; however, there was often a mismatch between the targeted levels of impact with the outcomes evaluated.
- Consistency in the identification and measurement of key outcomes across the different levels of impact may increase the impact and sustainability of programmes.

## Background

Researchers, health policymakers, leaders, educators, and health-research collaboratives are becoming increasingly interested in effective ways to rapidly translate research into practice to improve healthcare delivery systems, and ultimately, health outcomes [1–3]. The field of implementation science has exploded over the past two decades, as more evidence has been generated to support strategies for translating research evidence into health practice and policy successfully, sustainably, and at scale [4]. Concurrently, there is growing recognition of the need to develop capacity within healthcare settings and among health professionals to promote evidence-based knowledge translation practices, and enable the consistent, timely and sustained use of research evidence in health practice [3, 5, 6]. This recognition has led to the emergence of education programmes in the field of implementation and dissemination science; many of which have been led by universities and target academic researchers [7, 8]. Few education programmes have specifically focused on developing knowledge translation skills in health professionals [4]. Developing the capacity and capability of healthcare services and health professionals to adopt, adapt, and implement research evidence is critical to the sustainability of healthcare delivery systems [3, 5]. For this paper, the term capacity is defined as the readiness of and access to resources needed for individuals and organisations to engage in knowledge translation. Capability is defined as individuals’ knowledge and skills required to engage in translation practice [9, 10].

Initiatives such as the establishment of research translation centres, academic health science centres and clinical research networks have also sought to drive integrated evidence-based healthcare delivery [11]. Investment has been made in the strategic implementation of roles such as embedded researchers [12], knowledge brokers [2, 13], mentors [14], and implementation support practitioners [3], in a bid to support the active, timely and sustained translation of research in healthcare settings. The existing evidence supporting the implementation, outcomes and sustainability of these, and other strategies, to promote the translation of research into healthcare practice, has not been reviewed systematically.

This review was undertaken as part of a broader programme of work to promote the rapid translation of research knowledge into rural and regional healthcare settings. Currently, published reviews of strategies to build knowledge translation capacity have focused predominantly on education, training and initiatives led by academic institutions and targeting either academic researchers or a mix of researchers and health professionals [4, 8]. Other reviews have focused on programmes to develop evidence-based practice knowledge, skills and capabilities for health professionals to conduct practice-based research [15, 16]. Another published review investigated the accessibility of online knowledge translation learning opportunities available for health professionals [17]. This current review aims to fill the gap in the literature by scoping the evidence on programmes that aim to build capacity and capability within settings in which healthcare is delivered to patients or consumers (healthcare settings) and in health professionals, to implement research in practice.

A search of Cochrane Database of Systematic Reviews, Joanna Briggs Institute’s (JBI) Evidence Synthesis, PROSPERO and Google Scholar for reviews of knowledge translation capacity and capability building programmes and models in healthcare settings, yielded no existing or planned reviews. The decision to undertake a scoping review, rather than a conventional systematic review, was based on three key factors: (1) the heterogeneity evident in knowledge translation capacity and capability building programmes and models implemented in healthcare settings; (2) the absence of an existing synthesis of evidence for knowledge translation capacity and capability building programmes delivered in healthcare settings or for health professionals and (3) the need to identify the gaps in knowledge about these types of programmes [18].

This scoping review aimed to scope the literature describing programmes or models designed to build capacity and capability for knowledge translation in healthcare settings, and the evidence supporting these programmes and models. The specific review questions were:

1. What models or approaches are used to develop knowledge translation capacity and capability in healthcare settings?
2. How are the models and approaches to building knowledge translation capacity and capability funded, and the efforts sustained in healthcare settings?
3. How are these models or approaches evaluated and what types of outcomes are reported?

## Methods

This review used the JBI scoping review methodology. Search terms were developed for population, concept and context (PCC). The review questions, inclusion and exclusion criteria and search strategies were developed in advance (Additional File 1 Scoping Review Protocol). The review is reported in line with the Preferred Reporting Items for Systematic reviews and Meta-Analyses (PRISMA) extension for scoping reviews (Additional File 2 PRISMA-ScR checklist [19].

### Search strategy

The JBI three-step search strategy was applied. The researchers identified a set of key papers based on their knowledge of knowledge translation capacity and capability building programmes. These papers were used to identify key search terms. In consultation with the research librarians (FR and JS; see “Acknowledgements”), the research team conducted preliminary scoping searches to test the search terms and strategy and refine the final search terms. A tailored search strategy using the search terms was developed for each academic database (Additional file 3 Search Strategy).

Academic databases searched included Ovid MEDLINE, CINAHL, Embase and PsycInfo. Selected grey literature platforms, based on the researchers’ knowledge of relevant websites and organisations, were searched. Where larger search yields were observed (e.g. via Google and Google Scholar), the first 250 items were reviewed (Additional file 4 Grey Literature Searches). The final research database searches were conducted on 30th December 2022 by a researcher with extensive systematic literature searching experience (EW) in consultation with the research librarians. Grey literature searches were conducted on 15th March 2023. Searches of the reference lists of included records and forward citation searches were undertaken.

### Inclusion criteria and exclusion criteria

Literature was selected according to predefined inclusion and exclusion criteria developed using the PCC framework (see Table 1). Research education or capacity-building programmes delivered to qualified health professionals, working in healthcare settings in high-income countries (HIC) as defined by the Organisation for Economic Co-operation and Development (OECD) [20], were included. The HICs criteria was included to introduce a level of homogeneity around the broader resource contexts of the study populations [21]. No date limits were applied, and all types of literature published up to 30 December 2022 were included. Literature published in English only was included, due to resource limitations.

**Table 1:** Inclusion and exclusion criteria

	**Inclusion criteria**	**Exclusion criteria**
Population	Health workers; health professionals; health programme managers; clinicians; practitioners	Undergraduate students; postgraduate students and practitioners not working in a healthcare setting
Concept	Research translation and implementation skills Capacity-building strategies including education and training, short courses, mentoring, dedicated role (e.g. knowledge broker) or resource, embedded or implementation support practitioner, or research partnerships, networks, or collaborations	General research skills; implementation strategies for specific projects with no evidence of sustained capacity or capability building strategies Education part of tertiary qualification; not focused on building capacity or capability in health settings Programmes or theoretical frameworks that have not yet been implemented
Context	Targeting participants or people in healthcare settings* HICs	Universities, research centres, government, general community Programmes and strategies implemented in low- and middle-income countries

### Study selection, quality appraisal and data extraction

Citations were imported into Covidence (Veritas Health Innovation, Melbourne, Australia) for screening. Titles and abstracts were screened independently by two reviewers, with conflicts resolved by a third independent reviewer. Similarly, full texts were reviewed by two researchers and the reasons for exclusion were documented (Additional file 5 Excluded Studies). Data were extracted from the included texts by two independent researchers. All texts were reviewed by a second researcher to ensure the accuracy and consistency of data extraction. Formal quality appraisal was not undertaken as part of the scoping review, in line with this methodology [18].

Extracted data were tabulated and results were synthesised using a descriptive approach guided by the review objectives. The distinction between capacity and capability building strategies described in the papers was not drawn or analysed as part of this review. Outcomes measured and reported in the papers were synthesised descriptively as guided by the review objectives, the scoping review methodology and drawing on Cooke’s framework [22]. Although Cooke’s framework was initially developed for evaluating research capacity building, the four structural levels of impact are also relevant to informing and evaluating approaches to build capacity for research building for *impact* on health practice [23]. Sustainability, although not included as a key concept in the initial database searches, was considered in relation to both programme funding and the maintenance and spread of the programme outcomes [24]. Sustainability features were identified throughout the data extraction and synthesis processes.

## Results

**Fig. 1:**
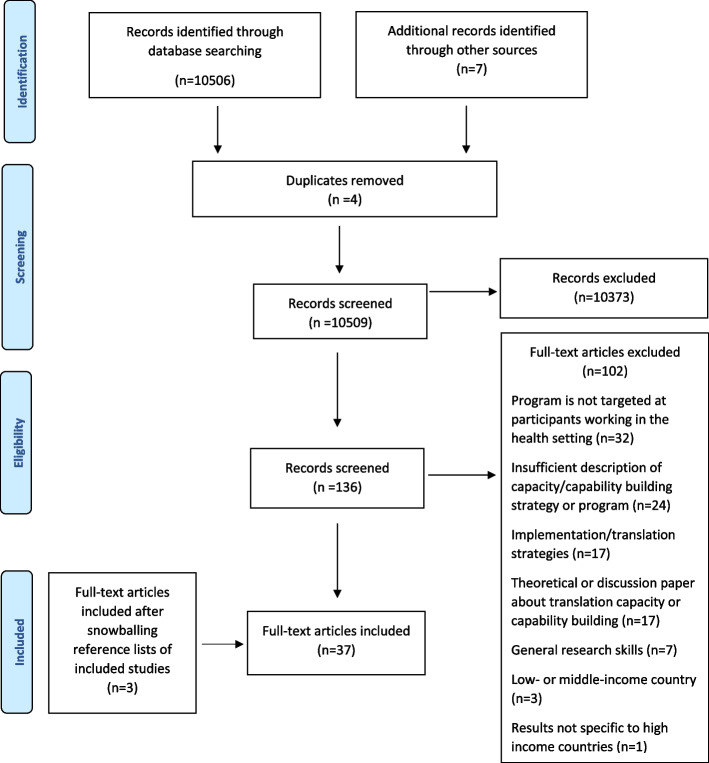
PRISMA flow diagram

Of the 10,509 titles and abstracts that were screened, 136 were included for full text screening. Of these, 34 met the inclusion criteria and the reasons for exclusion of 102 articles are shown in Fig. 1. Through citation search of the initial set, which involved hand-searching of reference lists, and forward searching of citations, an additional three papers were identified [25].

### Knowledge translation capacity and capability building programme delivery

A total of 37 papers, examining 34 knowledge translation capacity and capability building programmes were included in this review. The summary of the knowledge translation capability building programmes and their characteristics are shown in Table 2. Programmes were delivered in Australia [6, 26–38], Canada [39–47], England [48–52], the United States of America [53–55], Sweden [56, 57], Scotland [58], Saudi Arabia [59] and in multiple countries [11, 60] and were implemented from 1999 to 2021. Programmes tended to target a mix of health and research professionals; however, some targeted specific groups including allied health [26, 27, 29, 32, 35–37, 55], nurses [34, 45, 49], doctors [59], managers [57] and cancer control practitioners [53].

**Table 2:** Knowledge translation capability building programme characteristics

Citation	Name of programme and country of implementation	Aims of programme	Funding	Capability building strategy/ies	Number (if stated) and type/s of professions involved and	Recruitment or engagement method	Programme description	Setting of implementation	Pedagogical principles or capability building theory
Astorino, 2022 [53]	Cancer Control Implementation Science Base Camp (CCISBC) USA	• Build capacity for co-creation between cancer control practitioners and researchers	• Government health department (Centre for Disease Control)	• Workplace training/education	8 Cancer control practitioners	• Teams recruited via three videos which explained what implementation science is, how to assemble a team, and how to recruit partners for your team • Aimed to select teams from diverse geographic regions and historically excluded populations	• Curriculum included slide decks delivered by 6 2-person teams (1 implementation researcher and 1 cancer control practitioner), interactive questions, companion guide and case studies • 8 learning domains: Introduction, assess context, finding evidence, adapting evidence, implementation strategies, facilitating, evaluation, sustainability	• Health service or organisation	• Didactic learning • Experiential learning (case studies) • Peer/ social/ collaborative learning
Bennett, 2016 [26]; Eames 2018 [27]	Knowledge translation (KT) capacity-building programme for occupational therapy clinicians Australia	• Build KT capacity within an occupational therapy department by considering the barriers and enablers to the use of KT identified by clinicians • Contribute to a culture change within the department	• Government health department (Queensland Health/ state government)	• Co-design knowledge translation capability building programme • Workplace training/education • Support role	52 Allied health	• All occupational therapists working at one hospital were recruited and involved	• Programme incorporated multiple strategies such as educational outreach, case studies, identifying time blocks for KT, mentoring, leadership, communication, documentation, resources development, funding a KT champion, KT goal-setting, KT reporting strategies • Education provided by 2 academics with knowledge and experience of KT theory and practice, via 3 X 1-h sessions at the beginning and 1 1-h refresher session at the 12-month mark • Team-based mentoring provided by researchers (between 3–6 X 1-h sessions) • Clinical team leaders set the expectation for KT within their teams and the department director reinforced the importance of KT through regular communication	• Health service or organisation	• Experiential learning (case studies) • Mentoring • Peer/ social/ collaborative learning • Research or knowledge translation/ implementation theory
Black, 2021 [39]	Knowledge Translation (KT) Challenge Canada	• Build capacity to move research evidence into health practice	• Research institute (Providence Health Care Research Institute)	• Workplace training/education • Support role • Funding for knowledge translation/ implementation	185 Mixed professionals (allied health, nursing, physicians, nurse practitioner, and other)	• Letter of intent with manager support and endorsement	• Multi-component 3-year implementation support programme that involves training, funding, peer review, and mentorship • Teams attended 2 half-day workshops focused on developing an implementation plan, evaluating effectiveness of implementation and practice change • Teams were also supported to find a mentor within their clinical area and had access to online resources • Teams awarded $5000	• Health service or organisation	• Didactic learning • Experiential learning • Mentoring
Christensen, 2017 [55]	Knowledge Translation (KT) Programme USA	• Support organisational goals of using evidence to guide patient care and producing new knowledge • Address barriers to KT by engaging small groups to participate in the development or modification of guidelines to implement in local settings	• Health service	• Support role • Workplace training/education	66 Allied health	• All clinicians required to engage in at least one level of KT: journal club, clinical outcome group (COG), or research • Clinicians chose their level of participation	• Two positions (1) EBP coordinator developed KT processes, education and support for clinicians facilitates the development and implementation of guidelines in clinical practice and (2) Research coordinator engaged clinicians in research activity to generate new knowledge, through education, support developing research questions and protocols, grant writing, ethics approval, data collection, manuscript preparation • Journal clubs (entry level KT) summarised key articles to address a clinical research question • COGs investigated a clinician-identified research question and developed recommendations for local setting • Research created new knowledge to address local clinical questions •Clinicians provided 3–6 h per year to participate in KT •KT/EBP education (minimum 1 h) provided to all clinicians through in-services and brief written summaries	• Health service or organisation	• Didactic learning • Experiential learning • Mentoring • Peer/ social/ collaborative learning
Cooke, 2015 [48]	Collaborative priority setting (CPS) England	• Use collaborative priority setting to address the knowledge translation gap	• Research institute (National Institute for Health Research [NIHR])	• Research-practice collaborative (more than two entities)	Mixed professionals	• Collaboration and Leadership in Applied Health Research and Care (CLAHRC) provided the environment and resources for collaborative partnerships between researchers, clinicians, and health leaders	• CLAHRC initiated a process of CPS with researchers, clinicians, research theme leads, and health leaders/managers • Multiple methods used to support CPS; methods based on historical partnerships between researchers and clinicians; platforms for negotiation and planning, and formal methods of consensus	• Health service or organisation	• Peer/ social/ collaborative learning
Davies, 2017 [58]	Knowledge into Action (K2A) model Scotland	• Translate the K2A model into practice • Develop and implement tangible activities and outputs to support the librarian community across NHS Scotland in getting evidence into practice	• Health service (NHS Scotland)	• Workplace training/education • Support role • Research-practice collaborative (more than two entities) • Co-design knowledge translation capability building programme	Mixed professionals	• Not described	• Co-designed research implementation capability building programme • Coordinating processes for evidence search and synthesis • Co-development and implementation of model focussing on Knowledge broker network (librarians, information support officers, knowledge managers, educational facilitators and others), technology platform, capacity and capability development, defining knowledge gaps, sourcing and quality-assuring knowledge, creating, combining, and sharing knowledge, and research and evaluation •Supporting knowledge exchange between people and dissemination of knowledge (e.g. via communities of practice) •Delivering knowledge in actionable formats (e.g. care bundles, decision aids)	• Health service or organisation	• Didactic learning • Peer/ social/ collaborative learning • Research or knowledge translation/ implementation theory
Davis, 2020 [60]	King’s College London Implementation Science Masterclass (ISM) England	• Train those interested in the application of implementation science methodologies and techniques, irrespective of their professional background, where they fall on the career trajectory, or their expertise •Encourage collaborative work through developing a network of implementation scientists from diverse disciplines, professions, work settings, and socio-demographics	• Research institute (NIHR)	• University education programme	501 Mixed professionals (clinicians, managers, economists, policymakers, patients, epidemiologists from academic and healthcare settings)	• Participants enrol themselves in the annual training programme	• Annual course delivered face-to-face over 2 full days • Curriculum follows a 4-block structure, with each block delivered within a half-day session • Blocks cover the following broad thematic areas: (1) introduction to implementation science, (2) implementation theories and frameworks; (3) implementation research and evaluation methods and designs, and (4) specialist topics such as how implementation science relates to improvement science and knowledge mobilisation • Faculty represent multiple countries and research/practice backgrounds spanning all areas of implementation science	• University	• Didactic learning • Peer/ social/ collaborative learning
Dobbins, 2009 [40]	Knowledge Broker (KB) role Canada	•Promote integration of best available evidence into policy and practice-related decisions	• Research institute (Canadian Institutes of Health Research)	• Support role • Workplace training/education • Research-practice collaborative (more than two entities)	30 Public health programme managers	• One decision-maker from each organisation participating in an implementation project was randomised to one of three intervention groups	• KB implemented in a full time role for 1-year • KB developed knowledge translation and exchange interventions for groups of public health professionals • KB facilitated evidence-informed decision making (EIDM) knowledge and capacity by mentoring, providing individual support (email, telephone and site visits), group education (regional workshop, webinars), and encouraging managers to model EIDM-behaviours • Regional workshops present evidence, facilitate discussions of evidence, and identify implications for local programme and policy; provide opportunities for individual and collaborative EIDM problem-solving • Webinars provided professional development opportunities (e.g. the steps of the EIDM process: identify issue, retrieve evidence, identify implications for local policy and practice, implement evidence, evaluate outcomes)	• Health service or organisation	• Experiential learning • Mentoring • Peer/ social/ collaborative learning • Research or knowledge translation/ implementation theory
Dobbins, 2018 [41] Dobbins, 2019 [42]	Tailored knowledge translation intervention by knowledge brokers (KB) Canada	• Facilitate evidence-informed decision making (EIDM) in public health	• Research institute (Canadian Institutes of Health Research)	• Support role • Workplace training/education • Research-practice collaborative (more than two entities)	235 Public health practitioners, managers, directors, and medical officers	• Not described	• 2 KBs were employed for 22 months across 3 Canadian Public Health Departments (cases) to implement KT interventions • KBs facilitated EIDM, through a tailored approach of 1:1 mentoring, large and small group interactive workshops, and assisting in development of policies and procedures	• Health service or organisation	• Mentoring • Peer/ social/ collaborative learning • Research or knowledge translation/ implementation theory
Gerrish, 2010 [49]	Collaborations for Leadership in Applied Health Research and Care (CLAHRCs) South Yorkshire England	• Develop an innovative model for conducting applied health research and translating findings into practice to improve patient outcomes • Embed research and dissemination across the relevant geographical area • Increase needs-based research capacity focused on public health goals	• Research institute (NIHR)	• Research-practice partnerships (two entities) • Support role • University education programme	Nurses	• Not described	• CLAHRC South Yorkshire comprises numerous strategies to promote applied research informed by the research agenda of the local population • NHS and academic partnerships generate research activities in which nurses can participate, and collaborative research facilitate integrated KT • University course (MSc in Clinical Research) to enable students to develop clinical and health services research knowledge and skills implemented in 2009 • Matched funding with university partners to develop doctoral fellowships linked to research themes	• Health service or organisation	• Didactic learning • Experiential learning • Peer/ social/ collaborative learning
Gerrish, 2014 [50]	KT capacity development secondments England	• Increase KT capacity among clinical and academic nurses from partner organisations	• Research institute (NIHR)	• Support role	14 Mixed (clinical and academic nurses and dietitians)	• Not described	• Secondment model involving 7 clinical and 7 academic nurses/dietitians seconded from their employing hospital or university to work on KT initiatives • Part-time secondments ranging 9–24 months • Clinical secondees had clinical expertise and organisational knowledge to contribute to KT teams; academic secondees contributed evaluation skills	• Health service or organisation	• Experiential learning • Peer/ social/ collaborative learning
Greenhalgh, 2006 [51]	Master of Science in Knowledge Translation Online programme England	•Build boundary spanners’ explicit and tacit knowledge relevant to their own KT work	• Not stated	• University education programme	Mixed professionals (typically senior GPs, local postgraduate tutors, or service managers) from 16 countries and 17 disciplines	• Not described	• Part-time (10 h per week) online course structured around units that occur in a 7-week cycle • 2–3 weeks of independent learning at the start of each unit followed by 2 weeks of concentrated virtual seminars to provide opportunities for students to discuss initial ideas, focus, reflect on, and refine their ideas for their assignments • Further online discussion to enable students to actively construct knowledge, both through formal knowledge (e.g. posting a published article) and through the exchange of tacit knowledge (e.g. via stories about their own experiences or observations	• University	• Constructivism • Didactic learning • Experiential learning • Peer/ social/ collaborative learning
Haynes, 2020 [28]	Australian Prevention Partnership Centre Australia	•Produce innovative, internationally significant research in systems science, economics, evaluation, implementation science and communication •Develop new tools and methods for chronic disease prevention	• Not stated	• Research-practice partnerships (two entities)	Mixed professionals	• Not described	• Knowledge mobilisation partnership with 6 operational strategies including capacity and skills • Training delivered by experts in prevention and systems change • Training provides a forum for project groups to meet and address issues of common interest • Capacity and skill building initiatives include workshops, webinars and mentoring targeted at research and policy partners (including practitioners)	• Other setting	• Experiential learning • Mentoring • Peer/ social/ collaborative learning
Hitch, 2019 [29]	Lead Research Occupational Therapist Australia	• Provide leadership and vision around embedding research into OT practice at the service • Build research capacity in the use and generation of research as a complementary and simultaneous process to building capacity for knowledge translation	• Health service	• Support role	90 Allied health	• Not described	• Leadership position for knowledge translation in occupational therapy • Key deliverables for the position included completion and publication of research projects, formulation of a strategic plan to build research capacity and culture, formulation of a database of research activity at the service, and development of documentation and resources to support the ongoing sustainability of the position	• Health service or organisation	• Mentoring
Lizarondo, 2021 [30]	The JBI Clinical Fellowship Programme Australia	• Promote the use of evidence in clinical practice	• Not stated	•Workplace training/education •University education programme	284 Mixed professionals (nurses, doctors, allied health)	• Not described	• Clinical fellowship programme consisting of 3 phases • Phase 1 is an intensive 5-day training programme on evidence implementation, healthcare quality improvement and clinical leadership • In phase 2 fellows conduct an implementation project over 20 weeks in their practice setting • Phase 3 involves another 5-day intensive training to write and report the findings of their project • Fellows paired with an external facilitator/ mentor for support around evidence implementation methods	• Health service or organisation (predominantly)	• Experiential learning • Mentoring
Martin, 2022 [31]	Certificate in Health Science—Health Services Innovation Australia	• Increase capacity to implemented evidence-based methods and practice in healthcare by building individual skills • Build a critical mass of innovation social capital within the health service	• Health service	• University education programme	60 Mixed professionals (mostly senior clinicians)	• Students selected from health services from clinical and non-clinical roles based on their innovation and leadership skills	• Bespoke university award qualification and associated programme comprising 3 core components: delivery of post-graduate level units, student support services, and innovation implementation support • 4 university subjects are completed part-time over 2 years	• University	• Didactic learning • Research or knowledge translation/ implementation theory • Experiential learning
Mickan, 2022 [32]	Allied Health Research Fellows Australia	• Use knowledge brokering activities to engage research interested clinicians in research	• Government health department (Queensland Health/state government)	• Workplace training/education • Support role • Research-practice collaborative (more than two entities)	21 Allied health	• Research interested allied health clinicians volunteered to participate in or lead a project • Manager support to participate was required	• 3 research fellows facilitated allied health clinicians to participate in and lead clinical research projects over 12 months • Fellows used 10 knowledge brokering activities • Fellows assessed clinicians’ knowledge and skills, and then tailored specific guidance at the appropriate level and time, for each clinician for their projects • Fellows also guided clinicians to identify and engage with stakeholders, through preparatory and debriefing meetings, and made explicit the value of connecting	• Health service or organisation	• Mentoring • Research or knowledge translation/ implementation theory
Moore, 2018 [43]	Practicing Knowledge Translation course Canada	• Increase knowledge of how to use evidence and apply implementation science in healthcare settings • Build on knowledge translation activities by engaging with teams, ensuring opportunities for long term training and coaching, and tailoring to the needs of team members	• Not stated	• Workplace training/education	17 Mixed professionals	• Recruitment emails, ads in online forums and newsletters • Eligible participants included clinicians, healthcare managers, researchers, and policymakers	• Course for implementation practitioners delivered over six months through a 3-day workshop and 11 webinars • Training comprised activities, assignments, feedback, interaction with an implementation facilitator, access to resources, and social learning opportunities	• Health service or organisation	• Behaviour change theory • Didactic learning • Experiential learning • Research or knowledge translation/ implementation theory
Morrow, 2022 [6]	TRAining in evideNcebaSed ImpLementATion for hEalth (TRANSLATE) Australia	• Provide the project Implementation Lead an understanding of their role, practical strategies, and applied skills to promote implementation success	• Research institute (Cancer Institute NSW, Cancer Australia, and Australian Government Research Training Programme)	• Other training/ education (non-university, non-health workplace) • Support role	9 Mixed professionals (nursing, genetic counselling, clinical research, medical education)	• Implementation Leads were primarily recruited via liaison with site investigators and other hospital staff stakeholders • Aimed to recruit healthcare workers with a working knowledge of the Lynch Syndrome referral pathway, however other hospital staff with relevant experience were also considered	• 1-day face-to-face workshop for Implementation Leads • Training material was tailored according to 2 structured implementation approaches • Standard training content included an introduction to implementation science and specific instructions, practical activities, and tools for moving through the 7 phases of the implementation approaches • One trial arm also received additional Theoretical Domains Framework-guided content in the theory-based implementation arm • Workshops supplemented by teleconferences and centralised project team to provide additional support; participants received an electronic and hard copy toolkit	• Health service or organisation	• Behaviour change theory • Didactic learning • Experiential learning (case studies) • Peer/ social/ collaborative learning • Research or knowledge translation/ implementation theory
Mosedale, 2022 [33]	The Research Translation Projects (RTP) programme Australia	• Improve healthcare practice and/or policy by providing competitive grant funding to support projects that investigate efficiencies that can be delivered to WA Health while maintaining and/or improving patient outcomes • To facilitate clinical and academic collaboration	• Government health department (Western Australian Department of Health)	• Funding for knowledge translation/ implementation • Research-practice collaborative (more than two entities)	59 Funded projects led by health, academic, and consumer organisations	• Competitive funding programme • Collaboration between disciplines and institutions is a key priority for successful funding	• The RTP programme funds short-term research projects (2 years) to improve healthcare practice and/or policy by investigating potential efficiencies •Each grant recipient is required to submit a completion report to provide an account of objectives achieved, whether or not the programme/intervention was found to provide efficiencies to the health system and whether the funding had led to other benefits (such as changes in culture, capacity, new collaborations etc.)	• Health service or organisation	• Peer/ social/ collaborative learning
Mosson, 2019 [56]	Building Implementation Capacity (BIC) Sweden	• Build implementation capacity in teams of professionals, including their managers	• Small grant (Stockholm County Council)	• Workplace training/education • Research-practice collaborative (more than two entities)	159 Mixed professionals (teams of health care and social care work units)	• Case 1: Local health organisation initially contacted the researcher about building capacity. Senior manager decided all units should participate. Email sent to unit managers by health service senior managers • Case 2: Invitation sent to healthcare and social care organisations in the region via email to relevant mailing lists as well as the Research and Development Unit website	• 5 X workshops comprising short lectures, practical work, peer support, between-workshop assignments, feedback from workshop leaders, individual and group reflections, and workshop leaders’ boosting activities (emails, phone calls, and workplace visits) • All participating managers were invited to a separate workshop to clarify their role as implementation leaders • A 6-step systematic implementation method was used	• Health service or organisation	• Didactic learning • Experiential learning • Peer/ social/ collaborative learning • Research or knowledge translation/ implementation theory
Park, 2018 [44]	Foundations in Knowledge Translation (KT) Canada	• Enhance participants’ knowledge and self-efficacy in KT practice • Develop and implement a KT project	• Research institute (Canadian Institutes of Health)	• Workplace training/education	46 Mixed professionals (clinicians, managers, educators, researchers from different clinical settings)	• Programme advertised through two research foundations/ institutes • Emails sent to multiple health services and universities • Clinicians, researchers, health care managers, and policy makers were eligible to participate • Letter of support required from manager/ decision-making partners of all participants • Applicants needed to identify a healthcare/clinical challenge, its relevance and a summary of the evidence for implementation • 2 KT researchers reviewed each application and excluded those with insufficient evidence underpinning or lack of focus on applying research evidence	• Multi-component initiative including an initial in-person workshop, 2 in-person booster workshops held 6 months apart, an online learning platform, 2 years of mentoring/coaching, and printed/online educational materials • Pre-workshop focus groups to tailor content to learning needs • Content included KT science principles, KT funding, evaluation, and sustainability • Training facilitated by 3 KT researchers with expertise in KT practice	• Health service or organisation	• Didactic learning • Experiential learning • Mentoring • Peer/ social/ collaborative learning • Research or knowledge translation/ implementation theory • Self-efficacy theory
Plamondon, 2013 [45]	Nursing Research Facilitator (NRF) Programme Canada	• Strengthen nurses’ engagement in and use of research by building meaningful partnerships and cultivating a culture of curiosity among nurses and other healthcare providers	Research institute (Michael Smith Foundation for Health Research)	• Support role	50 funded projectsNurses	• Not described	• NRFs are nurses with a research background. Their role is to strengthen research culture, develop capacity, build partnerships, and provide a nursing voice for research across British Columbia • NRFs focus on organisation-wide applied health services research, and supporting opportunities for nurses to engage in research and knowledge translation	• Health service or organisation	• Mentoring
Proctor, 2019 [54]	Training in Implementation Practice Leadership (TRIPLE) USA	• Train clinical leaders and managers in implementation practice • Promote leadership skills and successful organisational change in regard to EBP implementation	• University (Fees subsidised by Centre for Dissemination and Implementation (CDI), Washington University)	• Workplace training/education	16 Mixed professionals (clinical managers, quality improvement coordinators, and programme directors)	• CDI sent emails to the CEOs of 8 organisations and invited them to nominate 2–3 clinical leaders of behavioural health programmes	• 3 in-person, half-day training sessions delivered 4 weeks apart, with optional conference calls with experts for coaching and support between sessions • Training was led by experts in behavioural health implementation and included lectures, individual and small group exercises, and reading assignments • Participants developed and piloted a small implementation project in their clinical setting •Networking among trainees promoted by sharing contact details	• Health service or organisation	• Didactic learning • Experiential learning • Mentoring • Peer/ social/ collaborative learning
Provvidenza, 2020 [46]	Knowledge Translation Facilitator Network (KTFN) Canada	• Foster better understanding and integration of KT into projects • Empower individuals to purposefully seek KT expertise and support	• Health service/ academic health science centre	• Workplace training/education • Research-practice collaborative (more than two entities)	33 Mixed professionals (healthcare providers, senior managers, students, trainees, research, family advisors)	• Interested individuals submitted an application including expected application of learnings • Participants selected if identified as actively leading strategic or integrated clinical research and/or education projects	• 6 sessions covered: introduction to KT, core KT principles, integrated versus end-of-grant KT, benefits of KT planning, implementation strategies, KT indicators for goal achievement/ evaluation, application via a KT simulation • Training delivered to small groups to optimise peer learning • KT specialist led the development of the curriculum with support from knowledge brokers for session facilitation • Participants received printouts of lectures, readings, and worksheets • Funding provided to cover participants’ backfill and training incidentals	• Health service or organisation	• Didactic learning • Experiential learning • Mentoring • Peer/ social/ collaborative learning
Richter, 2020 [57]	iLead Sweden	• Develop managers’ implementation leadership skills	• Small grant (AFA Insurance)	• Workplace training/education • Co-design knowledge translation capability building programme	52 (across 2 groups) Managers	• Self-nomination from defined groups of managers	• 5 half-day workshops covering introduction to the leadership and behaviour change models/theories, understanding and responding to employee reactions/resistance, analysing implementation target behaviour, creating action plans, adapting, measuring, and sustaining change • Programme goals and activities were co-designed with implementation and leaderships experts, 31 first line, and 9 senior-managers • Assignment work was conducted between workshops and peer and facilitator feedback provided during the workshops • Emails and feedback from colleagues occurred between workshops	• Health service or organisation	• Behaviour change theory • Experiential learning • Peer/ social/ collaborative learning
Robinson 2020 [11]	Research Translation Centres (RTCs) Australia and the UK	• Improve the integration of research, education, and healthcare • Accelerate the generation and translation of new evidence by fostering meaningful collaboration and integration between universities, health services and education providers • Generate research and education that is responsive to health service and community priorities	• Government health department • Research institutes (Australia: Medical Research Future Fund and UK: NIHR and other sources)	• Research-practice partnerships (two entities) • Research-practice collaborative (more than two entities) • Co-design knowledge translation capability building programme • Collaborative priority setting	12 RTCs	• Not described	• RTCs across Australia and the UK function differently depending on the characteristics of their partnering organisations and communities they serve • Common to each RTC is active participation in, and community centric integrated healthcare, education, and research • RTCs strive for meaningful and continuous engagement with stakeholders to better understand priorities and health risks and to collectively generate priority knowledge to improve health outcomes	• Health service or organisation	• Peer/ social/ collaborative learning
Sinfield, 2012 [52]	Collaboration for Leadership in Applied Health Research and Care (CLARHC)—LeichesteshireNorthamptonshire and Rutland (LNR) England	• Incorporate interprofessional education activities to reduce the second gap in translation (i.e. the long delay between conducting research and having an impact on clinical practice)	• Research institute (NIHR)	• Workplace training/education • Research-practice collaborative (more than two entities) • Support role	Mixed (doctors, nurses, allied health, and managers)	• Menu of courses was offered provided to the CLARHC NHS trusts to choose from	• CLAHRC is a clinical-academic partnership between a university and NHS trusts • Co-ordinators and fellow were embedded in NHS trusts • Co-ordinators and fellows assist research users to incorporate evidence in practice and policy decisions; promote a positive research culture within their organisation • Interprofessional workshops and e-modules to develop research implementation skills and knowledge around using research evidence to improve local policies and guidelines and evaluating healthcare services • A bank of resources, train the trainer, and e-learning strategies are in place to promote sustainability of the training opportunities • Knowledge exchange seminars and workshops between researchers, educators and clinicians, and managers across participating trusts	• Health service or organisation	• Didactic learning • Peer/ social/ collaborative learning
Thomson 2019 [47]	SUPPORT (Support for People and Patient-Oriented Research and Trials) KT Platform Canada	• Address Alberta’s SUPPORT stakeholder knowledge translation needs	• Research institute (Canadian Institutes for Health Research and Alberta Innovates)	• Other training/ education (non-university, non-health workplace) • Research-practice collaborative (more than two entities) • Co-design knowledge translation capability building programme	Mixed professionals	• Not described	• Multiple strategies to promote KT skills • Training workshops, webinars, lunch and learn sessions, conferences • KT Alberta community of practice to initiate and foster opportunities for capacity building and collaboration • KT consultation and support services to facilitate knowledge syntheses, and knowledge mobilisation and implementation • Co-design of KT strategies and practical guides	• Other setting	• Experiential learning • Peer/ social/ collaborative learning
Wahabi 2011 [59]	Innovative Teaching Workshop Saudi Arabia	• Build trainers’ skills in knowledge brokering and translation • Establish networks between evidence-based medicine experts and the end-users of that evidence (e.g. policy makers)	• Government health department (Course delivered via a university and supported by the Ministry of Health)	• University education programme	21 Medical doctors	• Prospective trainers selected from a group of family medicine physicians who had completed their residency and were board-certified • Priority given to those with prior teaching experience	• Train the trainer workshop to consolidate the skills and knowledge of participants with good baseline skills in evidence-based medicine • 5 workshops of between 3 and 5 h and 15 min duration • Topics covered included formulating an answerable question, hierarchy of evidence, introduction to KT, critical appraisal of evidence, reducing the knowledge to practice gap, barriers and enablers of KT • Workshops involved participant presentations and debate sessions	• University	• Debate • Didactic learning • Experiential learning • Peer/ social/ collaborative learning • Research or knowledge translation/ implementation theory
Wales, 2013 [34]	Facilitating change in clinical practice programme Australia	• Provide an overview of the programme for nursing staff with little experience of facilitation, undertaking projects underpinned by transformational practice development (tPD) methodology • Further enhance the expertise of facilitators presenting the programme using a co-facilitation model	• Not stated	• Workplace training/education	19 Nurses	• Invitation to attend the programme via expression of interest that included their reasons for undertaking the programme and the ways in which they believed their participation would benefit themselves and their unit/department •Support of their manager was required for full participation	• 1-year programme including 3 full-day workshops, active learning groups, and ongoing support using a co-facilitation model • Focused on strategies consistent with tPD, a methodology to change the culture and context of practice to develop sustainable person-centred and evidence-based workplaces	• Health service or organisation	• Experiential learning • Peer/ social/ collaborative learning • Self-efficacy theory
Wenke, 2018 [35]	Health Practitioner (HP) Research Fellow Australia	• Increase the research capacity of allied health professionals using dedicated research positions in healthcare organisations	• Queensland Health/state government	• Support role	29 Allied health	• Not described	• Dedicated research position implemented within a large regional health service to conduct and support practitioner-led research • Research fellows developed research infrastructure and strategic collaborations to build the research culture across the hospital and health service	• Health service or organisation	• Mentoring
Wilkinson, 2022 [36] Young, 2023 [37]	Knowledge Translation Support Service (KTSS) Allied Health Translating Research into Practice (AH-TRIP) Australia	• Provide accessible support for allied health professionals to promote impactful and sustained knowledge translation • Build capacity for KT (at an individual, group and organisational level) in the public health allied health workforce	• Queensland Health/state government	• Workplace training/education • Support role • Co-design knowledge translation capability building programme	9 (4 project teams) Allied health 148 (mentoring component) and 986 (at least one component) Allied health	• Invitation to participate via allied health professional (AHP) specific email lists with Queensland Health hospitals • Expressions of interest were screened to ensure appropriateness of the project	• 6-month multifaceted mentored KT training programme for AHPs working in a clinical capacity and undertaking a KT project in their setting • Online asynchronous training (webinars); support and networks (mentoring); AH-TRIP champions; showcase and recognition, and TRIP projects and implementation • AH-TRIP was co-designed with stakeholders; informed by theory and existing evidence • Monthly 1-h-long online facilitated mentoring group sessions • Case studies presented at each session representing two participant projects • Mentoring provided by a panel of 3 KT enthusiasts (KT experts and health service leaders)	• Health service or organisation	• Experiential learning (case studies) • Mentoring • Peer/ social/ collaborative learning • Self-efficacy theory
Wolfenden, 2017 [38]	Hunter New England Population Health (HNEPH) Research Partnership Australia	• Codesign and deliver evidence-based health services and conduct population health service delivery-focused research	• State government	• Research-practice partnerships (two entities) • Support role	Mixed professionals	• Not described	• Research-practice partnership between HNEPH Unit and University of Newcastle • Researchers are embedded in the health service delivery unit • An integrated governance structure oversees health service delivery and research initiatives • Senior researchers are also in health service management roles and senior health service managers lead research initiatives • Research capacity-building initiatives include PhD and postdoctoral research training	• Health service or organisation	• Experiential learning • Peer/ social/ collaborative learning

#### Strategies for building knowledge translation capacity and capability in health professionals and healthcare settings

Various capacity and capability building strategies were identified in the programmes. More than half of the programmes were described as using a combination of two or more strategies to build knowledge translation capability [6, 11, 26, 27, 30, 32, 33, 36–42, 46, 47, 49, 52, 55–58]. Programmes commonly involved targeted training and education for individuals and teams, delivered predominantly in the healthcare workplace, with few delivered in universities [31, 51, 59, 60], or other settings (e.g. partnership organisations) [28, 47]. Education was frequently employed in concert with other strategies such as dedicated implementation support roles [6, 26, 27, 32, 36–42, 46, 47, 49, 52, 55–58].

Other initiatives included strategic research-practice partnerships, typically between a health service and academic institution [11, 28, 38, 49], collaboratives (three or more research-interested organisations) [11, 32, 33, 40–42, 46–48, 52, 56, 58], co-designed knowledge translation capacity-building programmes with health professionals or health programme managers [11, 26, 27, 36, 37, 47, 57, 58], and dedicated funding for knowledge translation initiatives [33, 39]. The programmes reporting isolated strategies utilised education [31, 34, 43, 44, 51, 53, 54, 59, 60], a support role [29, 35, 45, 50] and research-practice partnerships [28].

The duration of the programmes varied significantly from 1-day workshops to upskill implementation leads [6] to comprehensive 3-year support programmes [39]. In some cases, programmes involving the implementation of a support role were described as ongoing [29, 35].

#### Pedagogical principles and theoretical frameworks

The pedagogical principles or learning theories underpinning the capability building programmes were rarely described explicitly, but rather were implied in the descriptions of the programmes. Many programmes purposefully made time in the curriculum for group work to foster connections with peers and promote social learning [6, 11, 26–28, 33, 34, 36–38, 40–42, 44, 46–60]. Further, experiential learning or “learning by doing” whereby participants applied their new knowledge and skills to a real-world project or knowledge translation initiative [61] was commonly described as a core component of capability building programmes [6, 26–28, 30, 31, 34, 36–40, 43, 44, 46, 47, 49–51, 53–57, 59]. Passive learning through didactic teaching (e.g. via lectures, seminars or webinars) was a common feature of education strategies [6, 31, 39, 43, 44, 46, 49, 51–56, 58–60]. Many programmes also incorporated individual or team-based mentoring with a more experienced knowledge translation specialist or researcher [26–30, 32, 35–37, 39–42, 44–46, 54, 55]. Behaviour change theory or techniques were referenced by a few studies [6, 43, 57]. Self-efficacy theory informed three programmes [34, 36, 37, 44]. Finally, one programme incorporated debate as a pedagogy [59].

#### Programme funding and sustained outcomes of the knowledge translation capacity and capability efforts

Sources of funding for the programmes included research institutes (e.g. Swedish Research Council, NIHR, Canadian Institutes of Health Research) [6, 11, 39–42, 44, 45, 47–50, 52, 60], government health departments (e.g. ministries or states responsible for health funding) [11, 26, 27, 32, 33, 53, 59], health services or academic health science centres [29, 31, 46, 55, 58], small grants [56, 57] and a university [54]. Five papers made no reference to a funding source [28, 30, 34, 43, 51].

Wenke [35] identified measures to promote the financial sustainability of the Health Practitioner Research Fellow role, including “Additional research and administrative funding, the use of technology and team based research” (p. 667). Proctor [54] identified the reliance on a single funding source for subsidising the TRIPLE programme as a threat to its sustainability. Similarly, Robinson [11] identified the 5-year funding cycles for Applied Research Collaborations as a factor undermining their sustainability. Gerrish [49] identified time limited funding of the Collaboration for Leadership in Applied Health Research and Care (CLAHRC) as a prompt to focus on “securing research grants and capitalising upon a range of opportunities for knowledge translation within a broader agenda focused on quality, innovation, productivity and prevention” (p. 223–224).

Moore [43] described potential mechanisms to ensure the sustainability of the Practicing Knowledge Translation programme, such as delivering online courses. Finally, Young [37] identified the adaptability of the AH-TRIP programme as a key sustainability feature, along with the establishment of a dedicated working group to conduct a formal sustainability assessment. Few papers explicitly described factors or mechanisms to sustain the efforts and outcomes of the knowledge translation capability building programmes. Hitch [29] described the development of a senior leadership position for knowledge translation in occupational therapy in which key deliverables included the development of documentation and resources to support the ongoing sustainability of the position. Similarly, Sinfield [52] described the development of a bank of resources housed on the CLAHRC website, a “train the trainer” model, and e-learning resources to sustain the capability building efforts.

Eleven programmes were guided by a knowledge translation theory or framework such as the Knowledge to Action (KTA) cycle [26, 27, 43, 44, 58, 59], the Dobbins (2002) Framework [40] and the National Collaborating Centre for Methods and Tools to frame the education programme [41, 42]. Martin [31] used the Consolidated Framework for Implementation Research (CFIR) to guide the implementation of the programme. Mickan [32] referred to the use of knowledge management theory, the linkage and exchange model and the social change framework to inform the functions of the knowledge brokers implemented in their programme. Morrow [6] used the Theoretical Domains Framework (TDF) and behaviour change theory in the development of their intervention. Mosson [56] used the principles of training transfer to inform their education programme.

#### Programme implementation level of influence and manager engagement

Programmes were categorised according to their implementation at four structural levels of impact in accordance with Cooke’s [22] framework: individual, team, organisational and supra-organisational. See Table 3 for the levels, definitions and citations. Interventions implemented at the individual or team level aimed to build the knowledge translation capacity of individuals and teams through increased knowledge, self-efficacy, research culture and engagement in knowledge translation. Programmes targeted at individuals included university courses [31, 51, 60], workplace training [44, 54, 57] and fellowship programmes [30]. Several programmes delivered training in a team environment to facilitate potential collaboration [26, 27, 36, 37, 39, 53]. Some larger-scale training interventions were implemented at an organisational level. For example, one study delivered workshops to teams across 35 units from different organisations [56]. Interventions aimed at this level most commonly took the form of dedicated research support roles, embedded within health organisations. These roles often involved educating interested health professionals through various means [29, 32, 41, 42, 45], strengthening research culture [45], engaging stakeholders [32], developing partnerships or collaborations [35, 45] and building research infrastructure [29, 35, 41, 42]. Other organisational strategies included secondments which provided health service staff with protected time to engage in knowledge translation endeavours [50]. Strategies implemented at the supra-organisational-level generally aimed to improve healthcare practice through collaboration, and strategies typically involved multifaceted initiatives of cross-organisational research collaborations such as CLAHRCs [48, 52] and Research Translation Centres [11]. In other cases, clinical-academic collaborations were fostered through a competitive funding initiative [33] and the development of communities of practice [58].

**Table 3:** Knowledge translation capability building programmes’ levels of impact

**Level of structural impact per Cooke’s ** [22]** framework**	**Definition**	**Citations**
Individual	Programmes that aim to build knowledge translation knowledge, skills and capability in individual health professionals or healthcare service staff	[30, 31, 34, 43, 44, 46, 51, 54, 55, 57, 59, 60]
Team	Programmes that take a team-based approach to building knowledge translation knowledge, skills and capability	[26, 27, 39, 53]
Organisational	Programmes that aim to build capacity and capability at the organisational level (e.g. by promoting sustainability of the programme or engaging participants from multiple levels of influence)	[6, 29, 32, 35, 40–42, 45, 50, 56]
Supra-organisational	Programmes that aim to build capacity and capability at the systems level (e.g. across healthcare networks or multiple healthcare organisations)	[11, 28, 33, 36–38, 47–49, 52, 58]

First line (middle) or senior executive managers were described as integral to many of the programmes to develop knowledge translation capacity and capability across the four levels of impact. Manager involvement was enacted in several ways: managers as programme participants [29, 38, 41–44, 46, 48, 49, 51–54, 56, 57, 60]; engagement or overt support of managers [26, 27, 32, 40, 50, 55]; letter of intent or support for team member participation [34, 39, 44]; managers were involved in the delivery of the strategy [38, 48] and co-design of the programme with managers [58]. In one programme, the manager needed to sign off to demonstrate their overt support for and was subsequently involved in the programme [56]. Several papers mentioned the presence and/or need for manager involvement or support in the outcomes or findings [11, 31, 35, 45, 49, 55]. One paper, describing a programme targeting doctors working in the family medicine context, did not explicitly refer to the involvement of managers; however, it did note that doctors in these settings also filled a managerial role [59].

### Programme and model evaluation

#### Evaluation methods

Twenty-seven programmes underwent some degree of formal evaluation, with defined aims and methods described to varying levels of detail in the papers (Table 4). The outcomes of the remaining seven programmes were described as the authors’ general reflections or learnings from some informal or not otherwise-described evaluation process [34, 38, 45, 49, 51, 52, 58]. Data collection methods used in the evaluations described included surveys [26–31, 39, 40, 43, 44, 46, 53–57, 59, 60], individual interviews [6, 11, 28, 30–32, 35–37, 43, 46–48, 50, 54, 56, 57], author reflections [26, 27, 34, 38, 45, 49, 51–53, 58–60], focus groups [26, 27, 30, 31, 35, 41, 44, 50], documentary analysis [28, 40, 42, 48, 55], attendance records [42, 43, 55], measured research outputs [29, 38] and observed changes to clinical guidelines, practice, or networks [38, 39, 55]. Twenty-four programmes were evaluated using multiple data collection methods.

**Table 4:** Evaluation and outcomes reported

Citation	Name of programme	Data collection methods	Evaluation sample size/s	Theoretical model utilised	Outcomes measured	Key findings
Astorino, 2022 [53]	Cancer Control Implementation Science Base Camp (CCISBC)	• Surveys: pre-post- programme • Author reflections	• 6	Kirkpatrick’s model	• Self-reported change in knowledge, skills, confidence, etc • Satisfaction / perceived quality of programme • Milestone achievement (e.g. implementation plan completed)	• Participants reported improvements in their knowledge of implementation science and its role in cancer care, how to implement evidence-based interventions to promote equity, implementation science terminology, sources of evidence-based interventions, and critical factors for sustaining an intervention • 33% “strongly agreed” and 66% “agreed” with the statement that they were satisfied with the content • 66% felt they could apply the plan created in team huddles to their work, and 91% felt they could put lessons learned from the training into their work • Feasible programme for collaborative learning between researchers and practitioners
Bennett, 2016 [26] Eames 2018 [27]	KT capacity-building programme for occupational therapy clinicians	• Survey • Focus groups • Author reflections/ observations • Surveys pre-/post- programme	• 52 (20 for entire duration) • 46 (baseline) and 39 (post programme)	Theoretical Domains Framework	• Barriers and enablers of knowledge translation • Self-reported changes in knowledge, skills, confidence, etc • Perceptions of organisational culture • Self-reported changes to clinical practice, guidelines, organisation policy, etc	• Enablers included “social/professional role and identity”, “reinforcement”, “social influence” and “beliefs about consequences” domains • Barriers at baseline included TDF domains of “attention, memory and decision processes”, “knowledge” and “environmental context and resources” • Significant improvements were seen in “knowledge”, “environmental context & resources”, “skills”, “beliefs about consequences”, “beliefs about capabilities”, and “memory, attention & decision processes” • Participants reported reading more clinical guidelines (10 vs. 17) and more participants reported using strategies to increase the use of recommended clinical practices • Participants agreed that KT had become part of the departments’ culture
Black, 2021 [39]	KT Challenge	• Surveys pre-/post- programme • Observed changes to clinical guidelines / practice / networks (etc.) • Document review	• Not stated	Not stated	• Attendance/ engagement with programme • Self-reported changes in knowledge, skills, confidence, etc • Milestone achievement (project completion) • Observed behaviour change • Barriers and enablers of knowledge translation	• Interest in programme remained steady with 4 cohorts taking part in the programme and 24 teams (185 healthcare professionals) funded to complete their projects • Participants reported statistically significant increases in their knowledge, confidence, and ability to implement practice change • 6/8 funded projects from the first cohort were successfully completed • 3/6 completed projects showed demonstrable practice changes across their respective practice areas • Reported challenges to engaging in KT included team member and manager turnover, communication with mentors, projects taking more time than anticipated, lack of support from key stakeholders for the practice change
Christensen, 2017 [55]	Knowledge Translation (KT) Programme	• Attendance records • Observed changes to clinical guidelines / practice / networks (etc.) • Documentary analysis • Survey post-programme	• 66	• Not stated	• Attendance/ engagement with programme • Observed behaviour change • Barriers and enablers of knowledge translation	• 100% staff engagement with at least one element of the KT programme • Clinician participation in development of local recommendations 68% • 9 topics with local recommendations produced by the over three years • Average compliance with 3 recommendations was 79% • Leadership support enabled KT activities and was vital to expanding the EBP and research coordinator roles
Cooke, 2015 [48]	Collaborative priority setting (CPS)	• Individual interviews • Documentary analysis	• 28	Not stated	• Participant experiences of programme • Perceptions of organisational culture	• CPS has the capacity to influence ongoing dialogue between researchers and clinicians through processes aligned with coproduction • CPS influenced the development of leadership skills in "theme leaders" who were a mix of NHS and academia-facing researchers • Key to the gains made by the CPS process were the resources and funding afforded by the Collaboration for Leadership in Applied Health Research and Care (CLAHRC) • Flexibility in the CPS process enabled responsiveness to evolving conditions in the health setting • CPS is time-consuming
Davies, 2017 [58]	Knowledge into action (K2A) model	• Author reflections /observations	N/A	N/A	N/A	• K2A had broad impacts on health librarians in NHS Scotland • Implementation of the model required the development of opportunities to build skills in areas such as summarising research findings, knowledge brokering and creating and supporting communities of practice
Davis, 2020 [60]	King’s College London Implementation Science Masterclass (ISM)	• Surveys post-programme • Author reflections /observations	• 323	Not stated	• Satisfaction and perceived quality of programme • Interest in programme/new applications	• Overall impression of the course was reported as “good” or “very good” by 92% of participants • Elements of the course including the learning objectives, pace and relevance or applicability of the content, and support provided were generally highly rated • 71% reported the course would have an impact on how they approached their future work • Faculty reflections identified the need to balance the educational delivery methods (i.e., didactic lectures and workshops); the need for different levels of ISM education (i.e. introductory vs advanced curriculum streams); the need for ongoing reflection and prioritisation of topic areas within the curriculum, and the need to provide advanced support and mentoring for some learners and their projects • Substantial growth of the course made it difficult to tailor to all delegates • Online options may address increasing demand for ISM training • Across 6 years participation has increased yearly from 40 participants in 2014 to 147 participants in 2019
Dobbins, 2009 [40]	Knowledge Broker (KB) role	• Documentary analysis	N/A	Not stated	• Engagement with programme	• Early needs assessments of participants and their organisations are vital to optimising the KB role • One-to-one contact to establish KB-participant relationship is crucial • A knowledge sharing mechanism or facilitated network is important to optimising participants’ limited time and resources and efficiently address their KT needs • KB roles take time to develop, and consequently capacity for evidence-informed decision making • Greater face-to-face interaction between the KB and participants may promote capacity for evidence-informed decision making • Research is needed to explore optimal preparation and training of the KB role, and key KB characteristics that promote effectiveness
Dobbins, 2018 [41] Dobbins, 2019 [42]	Tailored knowledge translation intervention by knowledge brokers (KB)	• Focus groups • Surveys pre-/post- programme • Documentary analysis	• Not stated • 606 (baseline) and 804 (post-programme) N/A	Not stated Not stated	• Perceptions of organisational culture • Self-reported changes in knowledge, skills, confidence, etc • Self-reported changes to clinical or implementation practice, guidelines, organisation policy, etc • Attendance/ engagement with programme	• In Case A, 48 staff were mentored individually or in small groups, and 33 participated in large group training • In Case B, 12 staff were mentored individually or in small groups, and 76 participated in large group training • In Case C, 17 staff were mentored individually or in small groups, and 49 participated in large group training • A statistically significant improvement in skills and knowledge was observed for all cases • There were no improvements in evidence-informed decision making behaviours except for those intensively involved in the programme • KT and evidence-informed decision making are complex processes and interventions need to be tailored to each specific context • Activities identified as important included policy and procedures plans, rapid evidence reviews, meetings with key individuals, and developing documents outlining the process for change • Resources, local community, culture, social and political issues all were important factors influencing evidence-informed decision making • Active engagement with the KB was key to participants’ development of evidence-informed decision making knowledge, skills, and behaviour
Gerrish, 2010 [49]	Collaborations for Leadership in Applied Health Research and Care (CLAHRCs) South Yorkshire	• Author reflections /observations	N/A	Not described	• Observed behaviour change	• CLAHRCs were developed around local health research priorities and provided opportunities for nurses and other frontline clinicians to engage in research • Without ongoing government funding for CLAHRCs, they will need to become financially self-sustaining (e.g. via research grants)
Gerrish, 2014 [50]	KT capacity development secondments	• Focus groups • Individual interviews • After action review group discussions	• 10 • 11 • 6 group discussions	Pluralistic evaluation design	• Participant experiences of programme • Self-reported changes in knowledge, skills, confidence, etc • Barriers and enablers of knowledge translation • Perceptions of organisational culture	• Clinical secondees acquired a range of KT skills • Academic secondees gained a better understanding of the healthcare contexts and improved their evaluation skills • Managerial support for secondees to optimise the balance between the KT and clinical/other roles is critical • Secondments facilitated capacity development and enhanced the diversity of skills in KT teams • Mentorship and support were important for secondees to perform optimally • Model benefited both the host and seconding organisations
Greenhalgh, 2006 [51]	Master of Science in Knowledge Translation Online programme	•Author reflections /observations	• N/A	Not stated	• Participant experiences of programme	• KT required tacit as well as explicit knowledge • Tacit knowledge had to be introduced into the organisation and integrated into the knowledge-creation cycle as well as acquired by individuals • A constructivist and collaborative approach to postgraduate education promoted the acquisition of tacit knowledge • Online environment provided a constructivist learning experience
Haynes, 2020 [28]	Australian Prevention Partnership Centre (“the Centre”)	• Individual interviews • Surveys pre-/post- programme • Documentary analysis	• 63 • Not stated	Not stated	• Satisfaction / perceived quality of programme • Attendance/ engagement with programme • New or expanded partnerships, collaborations, or networks	• Involvement in partnership facilitated sharing of ideas, collaboration, and communication • Engagement of policymakers influenced their uptake of research knowledge, methodologies, and resources • Partnerships grew over the Centre’s 5-year lifespan • Perceptions of leadership and engagement increased in some areas over time • Capacity-building activities were considered sufficiently frequent, varied, well-attended and well-received • Connections between partners not yet sufficient to form a co-produced prevention narrative
Hitch, 2019 [29]	Lead Research Occupational Therapist	• Surveys pre-/post- programme • Measured research outputs	• 42 (baseline) and 44 (post programme)	Not stated	• Self-reported changes in knowledge, skills, confidence, etc • Participant experiences of programme • Traditional research outputs • New or expanded partnerships, collaborations, or networks	• There was an increase in participation of occupational therapists in quality assurance and knowledge translation activities • There were no significant differences in the attitudes of occupational therapists toward EBP • Positive perception among Ots of the Lead Research Occupational Therapist role • EBP social networks showed more connections between clinicians, and less bottlenecks (where a single clinicians is the only point of contact between areas of the network) indicating an increase in the links between, and awareness of clinicians within the network • Key performance indicators were met over the first 3.5 years of the Lead Research Occupational Therapist position • 28 active research projects underway involving the active participation of 41 individuals • 10 clinician led articles published or accepted for publication with 5 more under review; 17 conference presentations, and 150,000AUD in research grants and fellowship funding
Lizarondo, 2021 [30]	Clinical Fellowship Programme	• Surveys post-programme • Individual interviews • Focus groups	• 43 • 16 • 9	Not stated	• Participant experiences of programme	• Implementation facilitation consisted of internal and external activities • External facilitation activities were undertaken by the external facilitators/mentors and included building trust, providing insight into clinical practice gaps and resources • Internal facilitation activities were undertaken by the fellows and included driving change in clinical practice, fostering group dynamics, and adapting evidence to the local context • Facilitators had a range of ideal characteristics including patience, communication skills, clinical background, approachable, skilled in evidence implementation
Martin, 2022 [31]	Certificate in Health Science – Health Services Innovation	• Focus groups • Individual interviews • Surveys post-programme	• 9 (students) • 9 (2 students, 3 university and 4 health service executive staff) • 28 (13 students, 8 managers, 7 control managers)	Consolidated Framework for Implementation Research	• Self-reported changes to clinical or implementation practice, guidelines, organisation policy, etc • Self-reported changes in knowledge, skills, confidence, etc • Barriers and enablers of knowledge translation	• The Health Services Innovation programme contributed to short-term improvements in individual and organisational capacity to implement evidence including the ability to identify knowledge gaps • Observed changes in capacity include increased connections and networks, use of a shared language, and use of implementation science methods • Executive support was a key enabler of sustained practice changes • Barriers to implementation related to the health service culture and readiness to adopt change
Mickan, 2022 [32]	Allied Health Research Fellows	• Individual interviews	• 3	Reflexive thematic analysis against knowledge brokering theory and practice	• Participant experiences of programme	• Three research fellows facilitated 21 clinicians’ participation in and leadership of clinical research projects over 12 months • Research fellows utilised all ten key knowledge brokering activities with each clinician • They used linkage and exchange activities for communicating and collaborating with key stakeholders, and tailored knowledge management products for individual’s engagement • They supported a broader learning journey through clarification and monitoring of individuals’ capabilities, motivation and contextual support for research engagement
Moore, 2018 [43]	Practicing Knowledge Translation course	•Surveys pre-/post- programme •Individual interviews (3 timepoints) •Attendance records	• 12 (baseline), 12 (3 months), 8 (6 months), and 6 (12 months) • 6 (3 months), 8 (6 months), and 6 (12 months)	Not stated	• Satisfaction / perceived quality of programme • Self-reported changes to clinical or implementation practice, guidelines, organisation policy, etc • Self-reported changes in knowledge, skills, confidence, etc • Attendance/ engagement with programme	• Participant satisfaction was high across all time points, mean scores ranged from 6.25 to 6.63 on a 7-point scale • Participants reported increased application of theories, models, and frameworks to their implementation projects • Increased self-reported knowledge and self-efficacy in core KT competencies • Increased self-efficacy in developing evidence-informed, theory-driven programmes • Attendance and completion of assignments decreased over the duration of the programme
Morrow, 2022 [6]	TRAining in evideNcebaSed ImpLementATion for hEalth (TRANSLATE)	• Individual interviews	• 9	Kirkpatrick’s model	• Satisfaction / perceived quality of programme • Self-reported changes in knowledge, skills, confidence, etc	• Participants reported overall satisfaction with the training and increased confidence in their ability to oversee trial implementation • Participants reported increased knowledge and skills related to evidence-based implementation however some participants found the theoretical concepts of behaviour change difficult to grasp • Teleconference support was valued • One externally recruited implementation lead noted the challenges of attempting to implement change as an “outsider” • Embedded implementation lead alleviated workload burden among other hospital staff
Mosedale, 2022 [33]	The Research Translation Projects (RTP) programme	• Document analysis	• 33 projects	Canadian Academy of Health Sciences’ framework for evaluation /Payback Framework	• Traditional and non-traditional research outputs • Self-reported changes to clinical or implementation practice, guidelines, organisation policy, etc • Self-reported changes in knowledge, skills, confidence, etc • New or expanded partnerships, collaborations, or networks	• The RTP programme resulted in 60 peer-reviewed publications, 122 conference presentations, and 4 other publications (educational resources, unpublished thesis, non-academic reports) • 10 projects reported media coverage • 6 PhD candidates used the projects for research contributing to their doctoral award • 19 projects gained additional research funding leveraged off the initial funding provided by the RTP programme • 14 projects reported a contribution to implementation of new local practice guidelines or policy, 8 projects reported making contributions to changes in policy or guidelines beyond the local setting • The programme led to increased research skills and knowledge, collaboration, partnerships, and networks
Mosson, 2019 [56]	Building Implementation Capacity (BIC)	• Surveys pre-/post- programme •Individual interviews	• 162 (baseline, programme participants), and 540 (baseline non-participants) and 98 (post-programme, [participants) and 189 (post-programme, non-participants) •36 (across 2 cases and 4 intervention groups)	Kirkpatrick’s model	• Satisfaction/ perceived quality of programme • Self-reported changes in knowledge, skills, confidence, etc • Self-reported changes to clinical practice, guidelines, organisation policy, etc	• Participants were satisfied with the BIC programme • Participants across all groups reported an increase in implementation knowledge • Most participants reported that they had applied what they had learned by enacting new implementation behaviour, however, they only partially applied the implementation method • Some changes to organisational context were reported in one group (e.g. increased readiness for implementation)
Park, 2018 [44]	Foundations in Knowledge Translation (KT)	• Surveys pre-/post- programme • Focus groups pre-/post- programme • Interviews	• 51 (baseline), 31 (6 months), 22 (12 months), 21 (18 months), 17 (24 months) • 85 focus groups and interviews with 2–3 participants in each across 4 timepoints	Promoting Action on Research Implementation in Health Services framework	• Self-reported changes in knowledge, skills, confidence, etc • Participant experiences of programme • Barriers and enablers of knowledge translation • Self-reported changes to clinical or implementation practice, guidelines, organisation policy, etc	• Participants’ self-efficacy in EBP, KT activities, and using evidence to inform practice all increased over time • Participants’ intention to use evidence in their work and their current use of research was high at baseline and did not change over time • Training facilitated participants to achieve their KT project objectives, plan their projects, and solve problems over time • Teams with high organisational capacity and commitment had upper managerial buy-in which resulted in secure funding and resource allocation • Participants reported using the knowledge and skills gained from the programme to integrate KT into grant applications with 5 project successful in obtaining funding • Participants applied their KT knowledge and skills to other projects and shared these with colleagues • Sustained KT practice was observed in 5 teams at the 2-year mark
Plamondon, 2013 [45]	Nursing Research Facilitator (NRF) Programme	• Author reflections / observations	Not stated	Not stated	• Barriers and enablers of knowledge translation	• The NRF programme provided facilitative support to over 50 funded research projects, led numerous workshops and journal clubs, and conducted more than 600 research-related consultations • NRFs offer transformative potential for influencing and implementing research in action because they are relatable to both clinicians and academics and are strategically positioned within health systems • Successful integration of NRFs rely on positioning and defining the role effectively and support of the NRF role by executive leaders • Challenges to integrating the NRF programme include organisational support and interest in the role, time needed to develop trust in and awareness of the role, and the NRFs ability to maintain credibility in both the research and clinical practice worlds
Proctor, 2019 [54]	Training in Implementation Practice Leadership (TRIPLE)	• Surveys pre-/post- programme (validated tools) • Individual interviews	• 13 • 9	Kirkpatrick’s model	• Satisfaction / perceived quality of programme / model / approach • Self-reported changes in knowledge, skills, confidence, etc	• 78.6% of participants rated the programme as having high levels of acceptability and appropriateness • Participants reported improvements in implementation leadership skills, knowledge and behaviour related to implementation practices • Implementation climate scales scores increased significantly, indicating improvements in organisational implementation culture • Participants were able to promote small changes within their organisations but were not able to implement a practice change within the evaluation timeframe
Provvidenza, 2020 [46]	Knowledge Translation Facilitator Network (KTFN)	• Surveys pre-/post- programme • Individual interviews	• 27 (baseline) and 18 (post-programme) • 28	Kirkpatrick’s model	• Self-reported changes in knowledge, skills, confidence, etc • Satisfaction / perceived quality of programme • Participant experiences of programme	• Participants reported increased confidence, knowledge, skills, and intention to use KT strategies following participation in training sessions • Participants were satisfied with the session content and presentation • Curriculum improvements were made in response to feedback including removal of homework, more variety in the sessions, new guest speakers, follow up knowledge burst emails, and emphasising practical aspects of the course
Richter, 2020 [57]	iLead	• Surveys pre-/post- programme • Individual interviews	• Group 1 – 15, 10, 8, 10 across four timepoints, Group 2 – 26, 23, 22, 22 across four timepoints, Group 1 general employees – 252, 160, 132 across three timepoints, Group 2 general employees—432, 313, 292 across three timepoints • 9	Kirkpatrick’s model	• Satisfaction / perceived quality of programme • Self-reported changes in knowledge, skills, confidence, etc • Self-reported changes to clinical or implementation practice, guidelines, organisation policy, etc	• Participants perceived the content and pedagogy of iLead to be relevant and high quality • Participants reported increased knowledge of implementation leadership • Participants who chose their implementation case had better experiences and outcomes of iLead • Organisational factors influenced managers’ experiences and the outcomes of iLead • More time to define the implementation case and the role of senior managers in supporting the participants is needed
Robinson 2020 [11]	Research translation centres (RTCs)	• Individual interviews	• 41 (representing 12 RTCs)	Not stated	• Barriers and enablers of knowledge translation • Participant experiences of programme	• Participants identified dissonant metrics and drivers between academic and healthcare sectors as a significant challenge • Participants deemed different models of leadership a crucial determinant of their success • Participants were unanimous on the importance of public and patient involvement however highlighted the need to better understand what makes for effective research co-production, and processes to support this • Participants agreed workforce development including a range of “global skills” and dedicated roles are needed to advance research translation • Collaboration was recognised as fundamental for RTCs, however several barriers were identified including incompatible funding cycles, differences in metrics, and high staff turnover
Sinfield, 2012 [52]	Collaboration for Leadership in Applied Health Research and Care (CLARHC) – Leichesteshire, Northamptonshire and Rutland	• Author reflections / observations	N/A	Not stated	• Satisfaction / perceived quality of programme	• Positive feedback from participants of workshops was reported • Success of the workshops and e-learning led to the development of a similar course on evaluating healthcare services • Knowledge exchange seminars provided opportunities for practitioners from primary and secondary care to meet and discuss practical solutions
Thomson 2019 [47]	SUPPORT (Support for People and Patient-Oriented Research and Trials) KT Platform	• Individual interviews	• 9	Not stated	• Self-reported changes to clinical or implementation practice, guidelines, organisation policy, etc • Satisfaction / perceived quality of programme	• The KT Platform was successful in assisting in KT with measurable changes in practice and improved patient outcomes • KT practitioners required ongoing support to develop confidence to undertake KT activities independently • Multidisciplinary team-based approaches to KT are needed
Wahabi 2011 [59]	Innovative Teaching Workshop	• Survey post-programme • Author reflections / observations	Not stated	Not stated	• Observed skill development • Satisfaction / perceived quality of programme	• Participants performed well in both the debate and knowledge translation project methods • Participants responded positively to debate as a pedagogical tool and its use in developing evidence-based medicine and KT skills • Participant perspectives of the KT project were positive, however some thought that it was outside of the scope of their role to advise about health policy • 98% agreed they would introduce debates as a method of teaching evidence-based medicine in the future • 52% agreed they would introduce knowledge translation projects as a method of teaching evidence-based medicine in the future
Wales, 2013 [34]	Facilitating change in clinical practice programme	• Author reflections / observations	Not stated	Not stated	• Satisfaction / perceived quality of programme • Participant experiences of programme • Self-reported changes in knowledge, skills, confidence, etc	• Facilitators were satisfied with how the programme was delivered • Small group work led to active participation, being productive, and sustained motivation • Diversity within the group enabled learning but required facilitators to be flexible and for a level of trust to be developed • Participants stated they gained skills facilitating change in clinical practice • Less experienced facilitators honed their skills and gained confidence • Dedicated time and venue for active learning groups and having manager support enabled attendance • Active learning groups were beneficial to learning • Gaining consensus on when to meet created tension and challenged group dynamic
Wenke, 2018 [35]	Health Practitioner (HP) Research Fellow	• Individual interviews • Focus groups	• 2 • 6	Not stated	• Observed behaviour change • Perceptions of organisational culture • New or expanded partnerships, collaborations, or networks • Barriers and enablers of knowledge translation	• Key outcomes included clinical and service improvements; enhanced research culture and skill development; development of research infrastructure (e.g. research committees) and formation of strategic research partnerships with universities and other research entities, and academic research outputs • Key barriers included time demands, challenges in participant recruitment, large geographical area to service, reduced awareness and accessibility of role, research position feeling alone, and physical resource constraints • Key enablers included leadership support, approachability of the research fellow, clear expectations, and clinician interest in research
Wilkinson, 2022 [36] Young, 2023 [37]	Knowledge Translation Support Service (KTSS) Allied Health Translating Research into Practice (AH-TRIP)	• Individual interviews • Document analysis	• 6 N/A	Theoretical Domains Framework RE-AIM framework	• Participant experiences of programme • Barriers and enablers of knowledge translation • Cost • Attendance/ engagement with programme • Satisfaction / perceived quality of programme • Milestone achievement (Showcase presentation)	• Barriers to enacting KT included lack of preparation, time pressures, and limited support within their projects • Enablers included internal motivation, access to an expert panel of mentors, and organisational and leadership support that included local champions, as well as the easy-to-use delivery platform helped to overcome them • Cost to deliver AH-TRIP was $AU197,595 per year to fund two dedicated positions to support the state wide programme, software licences, and direct in-kind costs (e.g. steering committee, working group meetings, webinar content development) • The AH-TRIP website with webinars and learning resources was viewed on average 944 times per month since its launch in March 2019 • The AH-TRIP champions network comprised more than 100 members who promoted AH-TRIP within their organisations • Telementoring supported 19 projects/teams across four cohorts; all telementees reported satisfaction with the programme • 49 teams submitted their project to the AH-TRIP annual showcase event • Strong organisational support for research and resources were key to organisations integrating AH-TRIP successfully
Wolfenden, 2017 [38]	Hunter New England Population Health (HNEPH) Research Partnership	• Author reflections/ observations • Observed changes to clinical guidelines / practice / networks (etc.)	N/A	Not stated	• Observed behaviour change	• The research-practice partnership maximised bidirectional knowledge exchange and facilitated immediate translation of research into practice • Dual leadership positions (across health delivery and university settings) enabled alignment in accountabilities • Co-contribution of resources between both organisations demonstrates mutual commitment to the partnership • The partnership has led to improved health system performance, the attraction of $40million in grant income since 2005, research translation impact, and higher degree completion • Time has been essential for the development of an integrated team of researchers involved in service delivery and research-engaged practitioners

#### Outcomes measured or described

Although the outcomes measured and reported varied significantly across the 34 programmes, all papers reported positive outcomes and the achievement of the programme objectives to varying extents. Outcome measures utilised in programme evaluations included participant self-reported improvements in knowledge, skills or confidence (etc.) [6, 26, 27, 29, 31, 33, 34, 39, 41, 43, 44, 46, 50, 53, 54, 56, 57], participant satisfaction with or perceived quality of the programme [6, 28, 34, 37, 46, 47, 52–54, 56, 57, 59, 60], participant experiences of programme [11, 29, 30, 32, 34, 36, 44, 46, 48, 50, 51], participant self-reported changes to clinical or knowledge translation practice, guidelines or organisation policy (etc.) [27, 31, 33, 41, 43, 44, 47, 56, 57], barriers and enablers of knowledge translation [11, 26, 27, 35, 36, 39, 45, 50, 55], attendance or engagement with programme [28, 37, 39, 40, 42, 43, 55], perceptions of organisational culture [26, 27, 35, 41, 48–50], observed or reported behaviour change (e.g. knowledge translation leadership development or changed clinical practice) [35, 38, 39, 49, 55], milestone achievement (e.g. implementation plans completed) [33, 37, 39, 53], new or expanded partnerships, collaborations, or networks [28, 29, 33, 35], traditional research outputs (e.g. papers, conference presentations, grants) [29, 33] and interest in programme or new applications [60].

#### Strengths and limitations of evaluation studies

Programme evaluations were strengthened by the inclusion of multiple outcome measures. Studies which incorporated multiple outcome measures often used a combination of self-reported outcomes or experience and more objective outcomes or observations such as milestone achievement [29, 36, 37, 39, 53, 55], observed behaviour change [35, 39, 44, 55] new or strengthened collaborations [29, 33], research outputs [29], observed skill development [59] and programme cost [36, 37]. Ten programme evaluations were informed by existing theoretical models including the Kirkpatrick Model [6, 46, 53, 54, 56, 57], the TDF [26, 27], the CFIR [31], Promoting Action on Research Implementation in Health Services framework [44], reflexive thematic analysis against knowledge brokering theory and practice [32] and the Canadian Academy of Health Sciences’ Framework for Evaluation and Payback Framework [33]. One programme used both the Reach, Effectiveness, Adoption, Implementation, Maintenance (RE-AIM) and TDF in two separate evaluations of the same programme [36, 37]. Evaluations tended to focus on short-term outcomes [6, 33, 46, 53, 59, 60]; however, there were examples of longer-term programme evaluation, as defined by data collected beyond 12-month post-programme delivery [26–29, 31, 32, 35–39, 41, 42, 44, 47, 49, 57].

There were some common limitations in the programme evaluations identified across the included papers. No studies measured health outcomes as a result of the programme. Several focused on only one outcome such as programme attendance or engagement [40], research outputs [38], perceptions or experiences of the programme [30, 32, 51], participant self-reported changes in knowledge, skills, confidence [6], barriers and enablers of knowledge translation [45], satisfaction or perceived quality of the programme [52]. One study did not identify any specific measurable outcomes [58]. Other commonly identified limitations were the use of self-reported outcomes only [26, 27, 33, 48, 50, 56, 57], and outcomes reported or measured among self-selected and likely more engaged and invested participants [11, 26, 28, 39, 44, 46, 47, 57]. Papers commonly described small sample sizes or low response rates in evaluations requiring participant involvement (e.g. surveys, interviews) [6, 11, 29–32, 37, 46, 47, 53]. Studies were often conducted at a single site or with a single cohort [29, 35, 41, 42, 44, 48], limiting the generalisability of the findings. Furthermore, programme evaluations were often limited by the inclusion of programme participants only in the data collection activities (e.g. [11, 26, 31, 36, 37, 47, 53]), i.e. there were no comparisons or controls. However, some programme evaluations engaged a broader range of relevant stakeholders to identify a more diverse range of outcomes at various levels of impact [28, 34, 35, 41, 42, 48, 50]. A notable example is the case study evaluation undertaken by Wenke [35] in which the relevant healthcare service executive director, incumbent holding the implementation support role, and six clinicians who had worked with the incumbent, participated in the evaluation. Similarly, Haynes’ [28] evaluation involved the chief investigators, members of the research network, and policy, practitioner and researcher partners.

There was an apparent lack of attention to the sustainability of programmes in the evaluations and there was only one evaluation which incorporated economic evaluation as part of the programme [37]. Further, measures of objective or observed behaviour change were included in only a few evaluations, including increased clinician engagement in research [35], the attraction of research funding [38], clinical service changes [35] and sustained knowledge translation practices post-programme participation [39, 44, 50, 55].

Although some studies investigated perceptions of organisational impacts such as research or knowledge translation culture [26, 27, 41, 42, 48–50] and barriers and enablers of knowledge translation [11, 26, 27, 35, 36, 39, 45, 50, 55], only one programme evaluation utilised a validated tool or approach to measuring organisational factors (the TDF) [26, 27]. Few studies used validated data collection tools to measure any types of outcomes [26, 27, 44, 54]. Only one evaluation included a control group in the data collection and analysis [31]. One study evaluated participant satisfaction and engagement with the programme only [60]. Poorly described or informal evaluation methods were identified in several papers [34, 38, 40, 45, 49, 51, 52, 55, 58, 59].

## Discussion

To our knowledge, this is the first scoping review of programmes designed to build capacity and capability for knowledge translation in healthcare settings. We sought to identify the models and approaches to building knowledge translation capability in healthcare settings, including the types of strategies used, the underpinning theories, funding sources, sustainability features, mechanisms of evaluation and the outcomes measured and reported. Our findings indicate that this is an area of increasing research interest and practice internationally [3]. We identified numerous types of strategies in place to promote knowledge translation capability in health settings, and an array of outcomes measured and reported in evaluations of these programmes.

Education was the most frequently described strategy and was delivered most often within health settings, followed by universities, and other organisations. Education was often delivered in concert with other strategies including implementation support roles, co-design of capability-building initiatives, funding for knowledge translation and strategic research-practice partnerships and collaboratives. This suggests that education is the cornerstone of knowledge translation capability building. It also points to the widespread recognition of the complexity of knowledge translation in practice [62–65] and the need to take a multifaceted approach to developing health professionals’ and healthcare service capacity and capability.

Programmes were implemented at four structural levels of impact [22]. Translating research in health practice requires the active involvement of and collaboration with various stakeholders [66, 67]; therefore, programmes aimed at team, organisational and supra-organisational level are more likely to see meaningful and sustained outcomes and impacts beyond the life of the programme and evaluation [4, 5, 68]. Social and experiential pedagogies [69], and mentoring featured prominently in the capability building programmes analysed as part of this review. Although didactic learning featured in many of the programmes in our review, this approach was complemented by either collaborative or experiential learning, or both. In contrast, Juckett et al. [4] found didactic coursework was a prominent feature within academic initiatives that aimed build advance knowledge translation practice.

Many programmes appeared to be dependent on time-limited funding or non-recurrent grants (government or philanthropic), with some only funded for discrete periods of time [36, 37, 45]. There were no references to ongoing funding sources to enable programme development, delivery or evaluation. This lack of certainty around funding and resourcing may undermine the continuity, quality, sustainability and impact of knowledge translation capability building programmes. Knowledge translation capacity-building programme leads can optimise the opportunities for ongoing funding by producing high-quality evaluations demonstrating impact on practice, and the alignment of their programmes with broader health policy agendas (e.g. promoting equity and quality in healthcare, and reducing inefficiencies) [70].

Although not always described explicitly as sustainability measures, there was evidence of these integrated in numerous programmes to maintain and further spread the impact of the programmes within the setting [24]. One of the sustainability features was the active engagement of managers in many of the programmes described in this review [26, 27, 29, 32, 34, 38–40, 42–44, 46, 48–58, 60]. This reinforces the recognition of the role of middle managers in supporting and mediating health practice changes, and in building capacity and positive attitudes toward knowledge translation within their teams [71, 72]. For organisations in which health professionals work independently, such as in medical and family practices, the middle manager role may be filled by the health professionals themselves [59]; therefore, strategies tailored to these settings and individuals are needed.

Both the content and implementation of several programmes were informed by knowledge translation theories or frameworks, which suggests a level of integrity in these programmes and the commitment of those developing and delivering the programmes to the theory and practices they seek to promote in participants. Furthermore, utilising evidence-informed implementation may promote the sustainability of the intervention in practice, and sustained outcomes and impact of the programme [73]. Several programmes were co-designed with end-users [26, 37, 38, 47, 57, 58], which not only increases the suitability of the programme to the local context but also increases a sense of ownership of the capability building programmes and strategies, and the potential to enhance sustainability [68, 74]. Other benefits of the early involvement of end-users in the development of capacity-building programmes in health settings include the integration of features and complexities reflective of the healthcare environment, and improved adoption and adaptation [75, 76]. This accentuates the need for future knowledge translation capacity and capability building programmes to be co-designed with end-users.

Overall, we found that programmes’ targeted levels of impact rarely corresponded with the outcomes measured in their evaluation. This highlights the need to develop standardised or at least streamlined frameworks that can be adopted by those leading the delivery or evaluation of programmes, to facilitate the planning and execution of appropriate evaluation. That is, if the programme targets the individual level, outcomes measured should relate to individuals (for example, self-reported improvements in knowledge, attitudes, satisfaction with programme) and if targeting the organisational level, outcomes related to research culture, the advent of new partnerships with research institutions or changes to organisational practice and policy, for example, should be measured. Similarly, programme evaluations rarely made clear the timeframe over which the outcomes were expected and measured. In one exemplary case, Young et al. [37] presented a programme logic which identified the programme components including inputs, activities and participant types, and linked these to the anticipated short-, medium- and long-term outcomes. The evaluation was then designed around these components and in reference to the RE-AIM framework. This points to the utility of programme logic in designing programmes and their evaluations.

Only one study, also Young et al.’s [37], explicitly referred to the absence of a dedicated evaluation budget as a limitation; however, this was likely the case for all programme delivery and evaluation teams, contributing to many of the identified limitations in the evaluations. Therefore, a standardised, theory-informed evaluation framework is needed to enable robust and consistent evaluation of multiple types of short-, medium- and longer-term outcomes, which correspond with the various levels of impacts [4, 6, 31, 32, 56]. This will enable more strategic programme implementation, make effective use of limited resources and provide for more illuminating programme evaluations to guide future capability building practice.

### Strengths and methodological limitations

This scoping review is strengthened by the systematic methods used. The involvement of a large team of researchers, with different levels of research and knowledge translation experience, representing different perspectives including experienced academic and knowledge translation researchers, those involved in developing and delivering knowledge translation capability building programmes, early career researchers and health professionals working in healthcare settings, in every stage of the review, enhanced the rigour of the study and the strength of our findings.

The main limitation of this review is the nature of the review topic and the existence of many synonyms and homonyms for several key concepts. The sheer breadth of relevant literature means the search strategy may not have included every relevant term, and therefore, may not have captured all eligible studies. Although formal quality assessment was not conducted, the limitations identified in the included papers and the absence of a formal evaluation in seven programmes indicate a generally low level of quality of the studies. This underlines the need for caution when interpreting and utilising the findings of this review. The settings in which the programmes were delivered were primarily larger health organisations with tiered managerial structures. Therefore, the findings, particularly as they relate to the role of and implications for middle managers, may not apply to contexts within which health professionals work independently (for example, physicians and family medicine doctors).

The databases searched did not include education research-specific databases, which may also have inadvertently excluded relevant papers from the search yield. The review of the programmes is limited to the strategies, characteristics, evaluation methods and outcomes as they were reported in the papers. It is likely that some of the papers did not include the details of all programme components and elements. This review is also limited by heterogeneity of the programmes with respect to the strategies described, outcomes measured, and findings reported. This diversity precluded the identification and application of a proxy measure of impact and subsequent comparison of the programmes. Nonetheless, as this scoping review aimed to map the programmes and strategies documented, the characteristics of the programmes, outcomes measured and reported, we were able to address the review questions.

### Implications for practice and future research

The review findings reinforce the need for knowledge translation capacity and capability building programmes to comprise multiple strategies working in concert to affect impact at the individual, team, organisational and supra-organisational levels. Practice-based pedagogies, collaborative learning and manager engagement are central to programmes to promote favourable outcomes. The review also highlighted several gaps in the literature. First, there is a need for more rigorous programme evaluations, which requires dedicated funding or resourcing. This could be supported by future research such as a focused systematic review on the outcomes and impacts of individual strategies (e.g. education), or structural levels of impact (e.g. team-level), to identify the most appropriate outcome measures and data collection methods. This will aid in simplifying programme evaluations and promote consistency across programmes. Furthermore, subsequent research could be undertaken to identify the presence and utility of any conceptual frameworks to guide capacity and capability building programme development, implementation and evaluation.

## Conclusion

There are a range of programmes that aim to develop knowledge translation capacity and capability in healthcare settings. Programmes tend to be multifaceted with education as the cornerstone, facilitate experiential and collaborative learning and target different levels of impact: individual, team, organisational and supra-organisational. All papers described successful outcomes and the achievement of programme objectives to some degree. Features to promote sustainability are evident; however, the sustainability of programmes and their outcomes and impacts may be threatened by the lack of commitment to long-term funding, and resourcing for rigorous programme evaluation. Indeed, the outcomes and impacts of these programmes are unclear and unable to be compared due to the often poorly described and widely inconsistent methods and outcome measures used to evaluate these programmes. Future research is required to inform the development of theory-informed frameworks to guide the use of methods and outcome measures to evaluate the short-, medium- and longer-term outcomes at the different structural levels, with a view to measuring objectively, the impacts on practice, policy and health outcomes in the longer term.

### Supplementary Information

**Additional file 1. **Scoping Review Protocol.

**Additional file 2. **Preferred Reporting Items for Systematic reviews and Meta-Analyses extension for Scoping Reviews (PRISMA-ScR) Checklist.

**Additional file 3. **Search Strategy.

**Additional file 4. **Grey Literature Searches.

**Additional file 5. **Excluded Studies.

## Data Availability

All data generated or analysed during this study are included in this published article and its supplementary information files.

## Abbreviations

AHP: Allied health professional
CFIR: Consolidated Framework for Implementation Research
CLAHRC: Collaboration for Leadership in Applied Health Research and Care
COG: Clinical outcome group
EBP: Evidence-based practice
EIDM: Evidence-informed decision making
HIC: High-income countries
JBI: Joanna Briggs Institute
KB: Knowledge broker
KT: Knowledge translation
KTA: Knowledge to Action
NHS: National Health Service
NIHR: National Institute for Health Research
OECD: Organisation for Economic Co-operation and Development
OT: Occupational therapist
PCC: Population, concept, and context
PRISMA: Preferred Reporting Items for Systematic reviews and Meta-Analyses
PRISMA-ScR: PRISMA extension for scoping reviews
RCB: Research capacity building
RE-AIM: Reach, Effectiveness, Adoption, Implementation, Maintenance
TDF: Theoretical Domains Framework
