# Supplementary material for: Models and approaches for building knowledge translation capacity and capability in health services: a scoping review
Source: Implement Sci. 2024 Jan 29;19:7. doi: 10.1186/s13012-024-01336-0 (PMC10823722; doi:10.1186/s13012-024-01336-0)
Supplement: Supplementary file 1 — Additional file 1. Scoping Review Protocol. [file 13012_2024_1336_MOESM1_ESM.docx]

# Additional File 1 - Scoping Review Protocol

The review questions, objectives and inclusion/exclusion criteria were developed by the research team. These formed a protocol for undertaking a scoping review using the Joanna Briggs Institute’s Methodology for Scoping Reviews [1].

## Review aim

To scope the evidence on models and approaches for building knowledge translation capacity and capability in health services.

## Review purpose

To inform the development and evaluation of a model to building research translation capacity and capability in health settings.

## Scoping review questions

1. What models or approaches are used to develop knowledge translation capacity and capability in healthcare settings?
2. How are the models and approaches to building knowledge translation capacity and capability funded, and the efforts sustained in healthcare settings?
3. How are these models or approaches evaluated and what types of outcomes are reported?

### Table 1 Inclusion and exclusion criteria

|  | **Inclusion criteria** | **Exclusion criteria** |
| --- | --- | --- |
| **Population** | Health workers; health professionals; health program managers; clinicians; practitioners | Undergraduate students; postgraduate students and practitioners not working in a healthcare setting |
| **Concept** | Research translation and implementation skills    Capacity building strategies including education and training, short courses, mentoring, dedicated role (e.g., knowledge broker) or resource, embedded or implementation support practitioner, or research partnerships, networks, or collaborations | General research skills; implementation strategies for specific projects with no evidence of sustained capacity or capability building strategies  Education part of tertiary qualification; not focused on building capacity or capability in health settings  Programs or theoretical frameworks that have not yet been implemented |
| **Context** | Targeting participants or people in healthcare settings*    HICs | Universities, research centres, government, general community    Programs and strategies implemented in low- and middle-income countries |

## Databases to be searched

Research databases for peer-reviewed literature: Ovid MEDLINE, CINAHL, Embase, and PsycInfo. Additional grey literature platforms: Google Scholar and Google.

## Data extraction

Data to be extracted will be input into two tables, and categorized under the following headings:

1. Citation; Name of program, Country of implementation; Year/s of implementation; Geographic region (as described in paper); Aims of program; Program scale; Capability building strategy/ies; Program funding source; Professions targeted; Number of participants; Recruitment/engagement method; Description of program, Setting of implementation, and Pedagogical principle/s or capability building theory/ies
2. Citation; Name of program; Evaluation data collection method/s; Sample size; Evaluation framework/s; Primary outcome measured; Secondary outcome measured; Tertiary outcome measured; Other outcome/s measured’ Key findings; Identified limitations
3. Program evaluation, data collection method and sample size; Primary outcome, secondary and other outcomes; Key findings.

#### Table 2. Search strategy: Ovid MEDLINE

| **#** | **Query** | **Results** |
| --- | --- | --- |
| 1 | Implementation Science/ | 1195 |
| 2 | Translational Science, Biomedical/ | 284 |
| 3 | Translational Research, Biomedical/ | 12967 |
| 4 | Information Dissemination/ | 19059 |
| 5 | (implementation adj3 (science or knowledge or research)).ti,ab. | 10213 |
| 6 | (translation* adj3 (science or knowledge or research)).ti,ab. | 22378 |
| 7 | (dissemination adj3 (science or knowledge or research)).ti,ab. | 3460 |
| 8 | (knowledge adj3 (transfer* or broker* or exchang*)).ti,ab. | 6570 |
| 9 | (improvement adj3 (science or research)).ti,ab. | 3316 |
| 10 | knowledge mobili?ation.ti,ab. | 173 |
| 11 | (practice adj3 chang*).ti,ab. | 18013 |
| 12 | 1 or 2 or 3 or 4 or 5 or 6 or 7 or 8 or 9 or 10 or 11 | 87533 |
| 13 | Capacity Building/ | 3289 |
| 14 | exp Education, Continuing/ | 62527 |
| 15 | exp Inservice Training/ | 29972 |
| 16 | Mentoring/ | 3481 |
| 17 | Curriculum/ | 84603 |
| 18 | Professional competence/ | 25068 |
| 19 | (capacity adj3 (build* or develop* or enhanc*)).ti,ab. | 28124 |
| 20 | (capability adj3 (build* or develop* or enhanc*)).ti,ab. | 4828 |
| 21 | 13 or 14 or 15 or 16 or 17 or 18 or 19 or 20 | 219240 |
| 22 | 12 and 21 | **4682** |

## References

1. Peters MD, Marnie C, Tricco AC, Pollock D, Munn Z, Alexander L, et al. Updated methodological guidance for the conduct of scoping reviews. JBI Evid Synth. 2020;18(10):2119-26.

2. The World Bank. Data for High income, OECD members, Upper middle income <https://data.worldbank.org/?locations=XD-OE-XT2021> [Available from: <https://data.worldbank.org/?locations=XD-OE-XT>.
