# Supplementary material for: Models and approaches for building knowledge translation capacity and capability in health services: a scoping review
Source: Implement Sci. 2024 Jan 29;19:7. doi: 10.1186/s13012-024-01336-0 (PMC10823722; doi:10.1186/s13012-024-01336-0)
Supplement: Supplementary file 3 — Additional file 3. Search Strategy. [file 13012_2024_1336_MOESM3_ESM.docx]

# Additional file 3 Search Strategy

**Aim:** To scope the evidence on models and approaches for building research translation capacity and capability in health services.

**Date:** 30/12/22

**Database:** Medline Ovid

| **#** | **Query** | **Results** |
| --- | --- | --- |
| 1 | Implementation Science/ | 1195 |
| 2 | Translational Science, Biomedical/ | 284 |
| 3 | Translational Research, Biomedical/ | 12967 |
| 4 | Information Dissemination/ | 19059 |
| 5 | (implementation adj3 (science or knowledge or research)).ti,ab. | 10213 |
| 6 | (translation* adj3 (science or knowledge or research)).ti,ab. | 22378 |
| 7 | (dissemination adj3 (science or knowledge or research)).ti,ab. | 3460 |
| 8 | (knowledge adj3 (transfer* or broker* or exchang*)).ti,ab. | 6570 |
| 9 | (improvement adj3 (science or research)).ti,ab. | 3316 |
| 10 | knowledge mobili?ation.ti,ab. | 173 |
| 11 | (practice adj3 chang*).ti,ab. | 18013 |
| 12 | 1 or 2 or 3 or 4 or 5 or 6 or 7 or 8 or 9 or 10 or 11 | 87533 |
| 13 | Capacity Building/ | 3289 |
| 14 | exp Education, Continuing/ | 62527 |
| 15 | exp Inservice Training/ | 29972 |
| 16 | Mentoring/ | 3481 |
| 17 | Curriculum/ | 84603 |
| 18 | Professional competence/ | 25068 |
| 19 | (capacity adj3 (build* or develop* or enhanc*)).ti,ab. | 28124 |
| 20 | (capability adj3 (build* or develop* or enhanc*)).ti,ab. | 4828 |
| 21 | 13 or 14 or 15 or 16 or 17 or 18 or 19 or 20 | 219240 |
| 22 | 12 and 21 | **4682** |
