# Supplementary material for: Models and approaches for building knowledge translation capacity and capability in health services: a scoping review
Source: Implement Sci. 2024 Jan 29;19:7. doi: 10.1186/s13012-024-01336-0 (PMC10823722; doi:10.1186/s13012-024-01336-0)
Supplement: Supplementary file 4 — Additional file 4. Grey Literature Searches. [file 13012_2024_1336_MOESM4_ESM.docx]

# Additional File 4 - Grey Literature Searches

| **Information source (e.g., name of organisation)** | **Website (url)** | **Search terms used and n results retrieved** | **Date of search** | **Citations retrieved (n)** | **Name of citations** | **URL** |
| --- | --- | --- | --- | --- | --- | --- |
| Google | http://www.google.com | Research translation capacity building  n= 93,000,000 | 15/03/2023 | 6 | Health Translation SA: Capacity BuildingCancer NSW: Translational Cancer Research Capacity Building Grant United States Agency for International Development: Capacity Building for Research Translation  Western Alliance: Research Translation Coordinators  Sydney Health Partners: Increasing Impact / Implementation Science  Laser Pulse Network: Embedded Research Translation | <https://healthtranslationsa.org.au/our-work/people/capacity-building/>  <https://www.cancer.nsw.gov.au/research-and-data/grants/grant-opportunities/translational-cancer-research-capacity-building-gr#:~:text=The%20Translational%20Cancer%20Research%20Capacity%20Building%20Grant%20aims%20to%20provide,with%20cancer%20across%20the%20state.>  <https://pdf.usaid.gov/pdf_docs/PA00ZC1B.pdf>  <https://www.westernalliance.org.au/research-translation-support/research-translation-coordinators/>  <https://sydneyhealthpartners.org.au/transl-imp/implementation-science/>  <https://laserpulse.org/embedded-research-translation/> |
| Google Scholar | <https://scholar.google.com.au/> | research translation capacity OR capability building OR development | 15/03/2023 | 0 |  |  |
| Google Scholar | <https://scholar.google.com.au/> | Knowledge translation capacity OR capability development  n= 4,180,000 | 15/03/2023 | 0 |  |  |
| Google Scholar | <https://scholar.google.com.au/> | Knowledge translation capacity OR capability building  n= 2,980,000 | 15/03/2023 | 0 |  |  |
| National Health Service | [The NHS website - NHS (www.nhs.uk)](https://www.nhs.uk/) | Research translation capacity building  n= 70 | 15/03/2023 | 0 |  |  |
| US Department of Health and Human Services | <https://health.gov/> | Research translation capacity building  n= 4 | 15/03/2023 | 0 |  |  |
| Australian Government Department of Health and Aged Care | <https://www.health.gov.au/> | Research translation capacity building  n= 237 | 15/03/2023 | 0 |  |  |
| Health Canada | <https://www.canada.ca/en/health-canada.html> | Research translation capacity building  n=7330 | 15/03/2023 | 1 | Canadian Institutes of Health Research: The Health System Impact Program | <https://cihr-irsc.gc.ca/e/51211.html> |
