# Supplementary material for: Models and approaches for building knowledge translation capacity and capability in health services: a scoping review
Source: Implement Sci. 2024 Jan 29;19:7. doi: 10.1186/s13012-024-01336-0 (PMC10823722; doi:10.1186/s13012-024-01336-0)
Supplement: Supplementary file 5 — Additional file 5. Excluded Studies. [file 13012_2024_1336_MOESM5_ESM.docx]

# Additional file 5 – Excluded studies

| **Reference** | **Reason for exclusion** |
| --- | --- |
| Berglund L, Tarantal A. Strategies for innovation and interdisciplinary translational research: removal of barriers through the CTSA mechanism. | Program is not targeted at participants working in the health setting |
| Brownson RC, Proctor EK, Luke DA, Baumann AA, Staub M, Brown MT, Johnson M. Building capacity for dissemination and implementation research: one university’s experience. Implementation Science. 2017 Dec;12:1-2. | Program is not targeted at participants working in the health setting |
| Bullock A, Morris ZS, Atwell C. Collaboration between health services managers and researchers: making a difference?. Journal of health services research & policy. 2012 Apr;17(2_suppl):2-10. | Program is not targeted at participants working in the health setting |
| Jacob RR, Gacad A, Padek M, Colditz GA, Emmons KM, Kerner JF, Chambers DA, Brownson RC. Mentored training and its association with dissemination and implementation research output: a quasi-experimental evaluation. Implementation Science. 2020 Dec;15(1):1-8. | Program is not targeted at participants working in the health setting |
| Kelly TH, Mattacola CG. Training and career development in clinical and translational science: an opportunity for rehabilitation scientists. Journal of Sport Rehabilitation. 2010 Nov 1;19(4):369-79. | Program is not targeted at participants working in the health setting |
| Romano M, Schnurr M. Mind the gap: Strategies to bridge the research-to-practice divide in early intervention caregiver coaching practices. Topics in Early Childhood Special Education. 2022 May;42(1):64-76. | Program is not targeted at participants working in the health setting |
| Baumann AA, Morshed AB, Tabak RG, Proctor EK. Toolkits for dissemination and implementation research: preliminary development. Journal of Clinical and Translational science. 2018 Aug;2(4):239-44. | Program is not targeted at participants working in the health setting |
| Brownson RC, Colditz GA, Dobbins M, Emmons KM, Kerner JF, Padek M, Proctor EK, Stange KC. Concocting that magic elixir: successful grant application writing in dissemination and implementation research. Clinical and translational science. 2015 Dec;8(6):710-6. | Program is not targeted at participants working in the health setting |
| Cheetham M, Wiseman A, Khazaeli B, Gibson E, Gray P, Van der Graaf P, Rushmer R. Embedded research: a promising way to create evidence-informed impact in public health?. Journal of Public Health. 2018 Mar 1;40(suppl_1):i64-70. | Program is not targeted at participants working in the health setting |
| Friedman DB, Escoffery C, Noblet SB, Agnone CM, Flicker KJ. Building capacity in implementation science for cancer prevention and control through a research network scholars program. Journal of Cancer Education. 2021:1-0. | Program is not targeted at participants working in the health setting |
| Kuchenmüller T, Chapman E, Takahashi R, Lester L, Reinap M, Ellen M, Haby MM. A comprehensive monitoring and evaluation framework for evidence to policy networks. Evaluation and program planning. 2022 Apr 1;91:102053. | Program is not targeted at participants working in the health setting |
| Libby AM, Ingbar DH, Nearing KA, Moss M, Albino J. Developing senior leadership for clinical and translational science. Journal of Clinical and Translational Science. 2018 Jun;2(3):124-8. | Program is not targeted at participants working in the health setting |
| Luke DA, Baumann AA, Carothers BJ, Landsverk J, Proctor EK. Forging a link between mentoring and collaboration: a new training model for implementation science. Implementation Science. 2016 Dec;11(1):1-2. | Program is not targeted at participants working in the health setting |
| Moore DW, Dilmore TC, Robinson GF. Advancing knowledge and research: developing a doctoral program in clinical and translational science. Clinical and translational science. 2011 Oct;4(5):359-62. | Program is not targeted at participants working in the health setting |
| Morrato EH, Rabin B, Proctor J, Cicutto LC, Battaglia CT, Lambert-Kerzner A, Leeman-Castillo B, Prahl-Wretling M, Nuechterlein B, Glasgow RE, Kempe A. Bringing it home: expanding the local reach of dissemination and implementation training via a university-based workshop. Implementation science. 2015 Dec;10:1-2. | Program is not targeted at participants working in the health setting |
| Padek M, Mir N, Jacob RR, Chambers DA, Dobbins M, Emmons KM, Kerner J, Kumanyika S, Pfund C, Proctor EK, Stange KC. Training scholars in dissemination and implementation research for cancer prevention and control: a mentored approach. Implementation Science. 2018 Dec;13(1):1-3. | Program is not targeted at participants working in the health setting |
| Proctor EK, Chambers DA. Training in dissemination and implementation research: a field-wide perspective. Translational Behavioral Medicine. 2017 Sep 1;7(3):624-35. | Program is not targeted at participants working in the health setting |
| Shelton RC, Dolor RJ, Tobin JN, Baumann A, Rohweder C, Patel S, Baldwin LM. Dissemination and implementation science resources, training, and scientific activities provided through CTSA programs nationally: Opportunities to advance D&I research and training capacity. Journal of clinical and translational science. 2022;6(1):e41. | Program is not targeted at participants working in the health setting 18 |
| Tramojntt C, Tejeda MZ, Guzanic J, Martini D, Korre M, Sokolovic M, Trigueiro H. 3 Development of regional Networks by the NNEdPro global centre for nutrition & health. | Program is not targeted at participants working in the health setting |
| Vogel AL, Hussain SF, Faupel-Badger JM. Evaluation of an online case study-based course in translational science for a broad scientific audience: Impacts on students’ knowledge, attitudes, planned scientific activities, and career goals. Journal of Clinical and Translational Science. 2022;6(1):e82. | Program is not targeted at participants working in the health setting |
| Pfund C, House SC, Asquith P, Fleming MF, Buhr KA, Burnham EL, Gilmore JM, Huskins WC, McGee R, Schurr K, Shapiro ED. Training mentors of clinical and translational research scholars: a randomized controlled trial. Academic medicine: journal of the Association of American Medical Colleges. 2014 May;89(5):774. | Program is not targeted at participants working in the health setting |
| Baumann AA, Carothers BJ, Landsverk J, Kryzer E, Aarons GA, Brownson RC, Glisson C, Mittman B, Proctor EK. Evaluation of the implementation research institute: trainees’ publications and grant productivity. Administration and Policy in Mental Health and Mental Health Services Research. 2020 Mar;47:254-64. | Program is not targeted at participants working in the health setting |
| Chambers DA, Pintello D, Juliano-Bult D. Capacity-building and training opportunities for implementation science in mental health. Psychiatry Research. 2020 Jan 1;283:112511. | Program is not targeted at participants working in the health setting |
| Duda MA, Riopelle RJ, Brown J. From theory to practice: an illustrative case for selecting evidence-based practices and building implementation capacity in three Canadian health jurisdictions. Evidence & Policy. 2014 Nov;10(4):565-77. | Program is not targeted at participants working in the health setting |
| Schultes MT, Aijaz M, Klug J, Fixsen DL. Competences for implementation science: what trainees need to learn and where they learn it. Advances in Health Sciences Education. 2021 Mar;26:19-35. | Program is not targeted at participants working in the health setting |
| Stevens KR, De La Rosa E, Ferrer RL, Finley EP, Flores BE, Forgione DA, Noel PH, Reistetter TA, Valerio-Shewmaker M, Wooten KC. Bootstrapping implementation research training: A successful approach for academic health centers. Journal of Clinical and Translational Science. 2021;5(1):e168. | Program is not targeted at participants working in the health setting |
| O’Brien MA, Makuwaza T, Graham ID, Barbera L, Earle CC, Brouwers MC, Grunfeld E. Lessons learned from a cancer knowledge translation grants program: results of an evaluation. Current Oncology. 2019 Aug;26(4):272-84. | Program is not targeted at participants working in the health setting |
| Oh AY, Emmons KM, Brownson RC, Glasgow RE, Foley KL, Lewis CC, Schnoll R, Huguet N, Caplon A, Chambers DA. Speeding Implementation in Cancer: The National Cancer Institute's Implementation Science in Cancer Control Centers. Journal of the National Cancer Institute. 2022 Oct 31:djac198-. | Program is not targeted at participants working in the health setting |
| Zepeda KG, Silva MM, Silva ÍR, Redko C, Gimbel S. Fundamentals of Implementation Science: an intensive course on an emerging field of research. Escola Anna Nery. 2018 May 14;22. | Program is not targeted at participants working in the health setting |
| Straus SE, Brouwers M, Johnson D, Lavis JN, Légaré F, Majumdar SR, McKibbon KA, Sales AE, Stacey D, Klein G, Grimshaw J. Core competencies in the science and practice of knowledge translation: description of a Canadian strategic training initiative. Implementation Science. 2011 Dec;6:1-7. | Program is not targeted at participants working in the health setting |
| Laser Pulse Network: Embedded Research Translation | Program is not targeted at participants working in the health setting |
| Canadian Institutes of Health Research: The Health System Impact Program | Program is not targeted at participants working in the health setting |
| Henderson A, Winch S. Staff development in the Australian context: Engaging with clinical contexts for successful knowledge transfer and utilisation. Nurse Education in Practice. 2008 May 1;8(3):165-9. | Insufficient description of capacity/capability building strategy or program |
| Hickman IJ, Cameron AE, McRae P, Wilkinson SA, O'Brien M, Kozica-Olenski S, Young AM. Feasibility and acceptability of a pilot knowledge translation telementoring program for allied health professionals. Internet Journal of Allied Health Sciences and Practice. 2021;19(4):17. | Insufficient description of capacity/capability building strategy or program |
| Auld R, Loppacher T, Rose S, Milat A, Penna A. Translational Research Grants Scheme (TRGS): a new approach to strengthening health system research capacity. Public Health Res Pract. 2018 Sep 27;28(3):2831818. | Insufficient description of capacity/capability building strategy or program |
| Sorkness CA, Pfund C, Asquith P, Drezner MK. Research mentor training: initiatives of the University of Wisconsin Institute for Clinical and Translational Research. Clinical and Translational Science. 2013 Aug;6(4):256. | Insufficient description of capacity/capability building strategy or program |
| Doherty S. Evidence‐based implementation of evidence‐based guidelines. International Journal of Health Care Quality Assurance. 2006;19(1):32-41. | Insufficient description of capacity/capability building strategy or program |
| Duncan C, Langlais S, Danyluk-Hall J, Simonson K. Knowledge translation: empowering health professionals to take the lead. Journal of Continuing Education in the Health Professions. 2008 Oct 1;28(4):282-3. | Insufficient description of capacity/capability building strategy or program |
| Donnelly C, Letts L, Klinger D, Shulha L. Supporting knowledge translation through evaluation: evaluator as knowledge broker. Canadian Journal of Program Evaluation. 2014 May 29;29(1). | Insufficient description of capacity/capability building strategy or program |
| Wales A, Boyle D. Scotland’s knowledge network: a progress report on Knowledge into Action. Scottish Medical Journal. 2015 Nov;60(4):155-8. | Insufficient description of capacity/capability building strategy or program |
| O’Brien MA, Grunfeld E. Building capacity in cancer knowledge translation through catalyst grants. Current Oncology. 2019 Feb;26(1):55. | Insufficient description of capacity/capability building strategy or program |
| Farr S, Decker CJ, Sales A, Spertus J. Novel Training In Implementation Science To Accelerate Evidence Into Practice. Circulation: Cardiovascular Quality and Outcomes. 2020 May;13(Suppl_1):A400-. | Insufficient description of capacity/capability building strategy or program |
| Bogaisky M, Vachna M, Jared MC, Cortes TA, Malik R, Lepore D, Zwerling J, Ehrlich AR. Facilitating Practice Change in Primacy Care Nurses: An Educational Intervention. In Journal of the American Geriatrics Society 2018 Apr 1 (Vol. 66, pp. S79-S79). | Insufficient description of capacity/capability building strategy or program |
| Grindell C. From MSK to TK2A: a musculoskeletal physiotherapists creative research capacity building journey. Physiotherapy. 2019 Jan 1;105:e193-4. | Insufficient description of capacity/capability building strategy or program |
| Drago, D.; Schwartz, L.; Luban, N.; Bocchino, J. Educating translational researchers using distance education.  Clinical and Translational Science. 2014; 7(3):224-225. | Insufficient description of capacity/capability building strategy or program |
| Allender S, Swinburn B, Foulkes C, Waters E, Gill T, Coveney J, Nichols M, Armstrong R, Sanigorski AD, Pettman T, Millar L. A new platform for increasing capacity in community based intervention: CO-OPS Mark II. Obesity Research & Clinical Practice. 2012(6):86-7. | Insufficient description of capacity/capability building strategy or program |
| Terasaki D. Mission, sensemaking, and tools: Exploring the capacity of Community Health Centers to implement evidence-based interventions. In Journal of Investigative Medicine 2011 Jan 1 (Vol. 59, No. 1, pp. 114-114). | Insufficient description of capacity/capability building strategy or program |
| Tran NT. Improving programme implementation through embedded implementation research (iPIER). In Tropical Medicine & International Health 2015 Sep 1 (Vol. 20, pp. 122-122). | Insufficient description of capacity/capability building strategy or program |
| Morrow A, Chan P, Hogden E, Taylor N. Building capacity from within-upskilling healthcare professionals to lead an evidence-based implementation approach. In Implementation Science 2020 Dec 17 (Vol. 15, No. Suppl 4). | Insufficient description of capacity/capability building strategy or program |
| Wine O, Spiers J, van Manen M, Burns KK, Vargas AO. Collaborative research, capacity building and knowledge translation development for research on adverse birth outcomes and the environment. Paediatrics & Child Health. 2018 May 18;23(suppl_1):e39-. | Insufficient description of capacity/capability building strategy or program |
| Kielly-Carroll C, Shaw T, Haines M, Dadich A, Sanson-Fisher R, Girgis A, Phillips J, Rankin N, Robinson T, Pointeaux C. Building the bridge from discovery-to-delivery: A community of practice in cancer implementation science. In Asia-Pacific Journal of Clinical Oncology 2015 Nov 1 (Vol. 11, pp. 107-107). | Insufficient description of capacity/capability building strategy or program |
| Kilbourne AM, Jones PL, Atkins D. Accelerating implementation of research in learning health systems: lessons learned from VA health services research and NCATS clinical science translation award programs. Journal of Clinical and Translational Science. 2020 Jun;4(3):195-200. | Insufficient description of capacity/capability building strategy or program |
| Health Translation SA: Capacity Building | Insufficient description of education intervention |
| Cancer NSW: Translational Cancer Research Capacity Building Grant | Insufficient description of education intervention |
| Western Alliance: Research Translation Coordinators | Insufficient description of education intervention |
| Sydney Health Partners: Increasing Impact / Implementation Science Academy | Insufficient description of education intervention |
| Barnsteiner JH, Reeder VC, Palma WH, Preston AM, Walton MK. Promoting evidence-based practice and translational research. Nursing Administration Quarterly. 2010 Jul 1;34(3):217-25. | Implementation/translation strategies used for a particular project |
| Légaré F, Borduas F, MacLeod T, Sketris I, Campbell B, Jacques A. Partnerships for knowledge translation and exchange in the context of continuing professional development. Journal of Continuing Education in the Health Professions. 2011 Jun;31(3):181-7. | Implementation/translation strategies used for a particular project |
| Petzold A, Korner‐Bitensky N, Menon A. Using the knowledge to action process model to incite clinical change. Journal of Continuing Education in the Health Professions. 2010 Jun;30(3):167-71. | Implementation/translation strategies used for a particular project |
| Cudd P, Fowler-Davis S, Evans L, Mawson S. Knowledge Exchange: selecting research opportunities through estimation. Studies in Health Technology and Informatics. 2015 Jan 1;217:736-43. | Implementation/translation strategies used for a particular project |
| Eljiz K, Greenfield D, Hogden A, Taylor R, Siddiqui N, Agaliotis M, Milosavljevic M. Improving knowledge translation for increased engagement and impact in healthcare. BMJ open quality. 2020 Sep 1;9(3):e000983. | Implementation/translation strategies used for a particular project |
| Elliott MJ, Allu S, Beaucage M, McKenzie S, Kappel J, Harvey R, Morrin L, Soroka S, Graham J, Harding C, Pinsk M. Defining the scope of knowledge translation within a national, patient-oriented kidney research network. Canadian Journal of Kidney Health and Disease. 2021 Apr;8:20543581211004803. | Implementation/translation strategies used for a particular project |
| Hurtubise K, Rivard L, Héguy L, Berbari J, Camden C. Virtual knowledge brokering: describing the roles and strategies used by knowledge brokers in a pediatric physiotherapy virtual community of practice. Journal of Continuing Education in the Health Professions. 2016 Jul 1;36(3):186-94. | Implementation/translation strategies used for a particular project |
| Moore JL, Carpenter J, Doyle AM, Doyle L, Hansen P, Hahn B, Hornby TG, Roth HR, Spoeri S, Tappan R, Van Der Laan K. Development, implementation, and use of a process to promote knowledge translation in rehabilitation. Archives of Physical Medicine and Rehabilitation. 2018 Jan 1;99(1):82-90. | Implementation/translation strategies used for a particular project |
| Puga F, Stevens KR, Patel DI. Adopting best practices from team science in a healthcare improvement research network: the impact on dissemination and implementation. Nursing Research and Practice. 2013 Jan 1;2013. | Implementation/translation strategies used for a particular project |
| Starmer AJ, Spector ND, West DC, Srivastava R, Sectish TC, Landrigan CP, Menon AA, Ali A, Allair BK, Allen AD, Almaddah N. Integrating research, quality improvement, and medical education for better handoffs and safer care: disseminating, adapting, and implementing the I-PASS program. The Joint Commission Journal on Quality and Patient Safety. 2017 Jul 1;43(7):319-29. | Implementation/translation strategies used for a particular project |
| Duhamel F. Translating knowledge from a family systems approach to clinical practice: Insights from knowledge translation research experiences. Journal of Family Nursing. 2017 Nov;23(4):461-87. | Implementation/translation strategies used for a particular project |
| Silk KJ, Walling B, Totzkay D, Mulroy M, Smith S, Quaderer T, Boumis J, Thomas B. Continuing medical education as a translational science opportunity for health communication researchers: the BCERP model. Health communication. 2020 Aug 23;35(10):1266-73. | Implementation/translation strategies used for a particular project |
| Rohweder C, Wangen M, Black M, Dolinger H, Wolf M, O'Reilly C, Brandt H, Leeman J. Understanding quality improvement collaboratives through an implementation science lens. Preventive medicine. 2019 Dec 1;129:105859. | Implementation/translation strategies used for a particular project |
| Schreiber J, Dole RL. The effect of knowledge translation procedures on application of information from a continuing education conference. Pediatric Physical Therapy. 2012 Oct 1;24(3):259-66. | Implementation/translation strategies used for a particular project |
| Holmes B, Scarrow G, Schellenberg M. Translating evidence into practice: the role of health research funders. Implementation Science. 2012 Dec;7(1):1-0. | Implementation/translation strategies used for a particular project |
| Happell B, Johnston L, Hill C. Implementing research findings into mental health nursing practice: exploring the clinical research fellowship approach. International Journal of Mental Health Nursing. 2003 Dec;12(4):251-8. | Implementation/translation strategies used for a particular project |
| Barker M, Lecce J, Ivanova A, Zawertailo L, Dragonetti R, Selby P. Interprofessional communities of practice in continuing medical education for promoting and sustaining practice change: a prospective cohort study. Journal of Continuing Education in the Health Professions. 2018 Apr 1;38(2):86-93. | Implementation/translation strategies used for a particular project |
| Albers B, Metz A, Burke K. Implementation support practitioners–a proposal for consolidating a diverse evidence base. BMC Health Services Research. 2020 Dec;20:1-0. | Protocol, theoretical or discussion paper about translation capacity or capability building |
| Arrington B, Kimmey J, Brewster M, Bentley J, Kane M, Van Brunschot C, Burns M, Quinlan K, Brownson RC. Building a local agenda for dissemination of research into practice. Journal of Public Health Management and Practice. 2008 Mar 1;14(2):185-92. | Protocol, theoretical or discussion paper about translation capacity or capability building |
| Beckett K, Farr M, Kothari A, Wye L, Le May A. Embracing complexity and uncertainty to create impact: exploring the processes and transformative potential of co-produced research through development of a social impact model. Health research policy and systems. 2018 Dec;16(1):1-8. | Protocol, theoretical or discussion paper about translation capacity or capability building |
| Bender BG, Krishnan JA, Chambers DA, Cloutier MM, Riekert KA, Rand CS, Schatz M, Thomson CC, Wilson SR, Apter A, Carson SS. American thoracic society and national heart, lung, and blood institute implementation research workshop report. Annals of the American Thoracic Society. 2015 Dec;12(12):S213-21. | Protocol, theoretical or discussion paper about translation capacity or capability building |
| Boland L, Kothari A, McCutcheon C, Graham ID. Building an integrated knowledge translation (IKT) evidence base: colloquium proceedings and research direction. Health Research Policy and Systems. 2020 Dec;18(1):1-7. | Protocol, theoretical or discussion paper about translation capacity or capability building |
| Kislov R, Waterman H, Harvey G, Boaden R. Rethinking capacity building for knowledge mobilisation: developing multilevel capabilities in healthcare organisations. Implementation Science. 2014 Dec;9:1-2. | Protocol, theoretical or discussion paper about translation capacity or capability building |
| Rapport F, Smith J, Hutchinson K, Clay‐Williams R, Churruca K, Bierbaum M, Braithwaite J. Too much theory and not enough practice? The challenge of implementation science application in healthcare practice. Journal of Evaluation in Clinical Practice. 2022 Dec;28(6):991-1002. | Protocol, theoretical or discussion paper about translation capacity or capability building |
| Barnett ML, Stadnick NA, Proctor EK, Dopp AR, Saldana L. Moving beyond Aim Three: a need for a transdisciplinary approach to build capacity for economic evaluations in implementation science. Implementation science communications. 2021 Dec;2(1):1-9. | Protocol, theoretical or discussion paper about translation capacity or capability building |
| Hwang S, Birken SA, Melvin CL, Rohweder CL, Smith JD. Designs and methods for implementation research: advancing the mission of the CTSA program. Journal of Clinical and Translational Science. 2020 Jun;4(3):159-67. | Protocol, theoretical or discussion paper about translation capacity or capability building |
| Inkelas M, Brown AF, Vassar SD, Sankaré IC, Martinez AB, Kubicek K, Kuo T, Mahajan A, Gould M, Mittman BS. Enhancing dissemination, implementation, and improvement science in CTSAs through regional partnerships. Clinical and translational science. 2015 Dec;8(6):800-6. | Protocol, theoretical or discussion paper about translation capacity or capability building |
| Gonzales R, Handley MA, Ackerman S, O'Sullivan PS. A framework for training health professionals in implementation and dissemination science. Academic Medicine. 2012 Mar 1;87(3):271-8. | Protocol, theoretical or discussion paper about translation capacity or capability building |
| O’Rourke N, O’Toole E. 192 Using implementation levers to translate evidence into practice. BMJ Evidence-Based Medicine. 2022 Jun 1;27(Suppl 1):A57-. | Protocol, theoretical or discussion paper about translation capacity or capability building |
| Javanbakhtian Ghahfarokhi R, Alavi M, Soleymani MR, Raeburn T, West S, Cleary M. Developing capacity around knowledge translation: A marriage of action and mindset. Issues in Mental Health Nursing. 2021 Sep 2;42(9):884-6. | Protocol, theoretical or discussion paper about translation capacity or capability building |
| Mickan S, Wenke R, Weir K, Bialocerkowski A, Noble C. Strategies for research engagement of clinicians in allied health (STRETCH): a mixed methods research protocol. BMJ open. 2017 Sep 1;7(9):e014876. | Protocol, theoretical or discussion paper about translation capacity or capability building |
| Haynes A, Rychetnik L, Finegood D, Irving M, Freebairn L, Hawe P. Applying systems thinking to knowledge mobilisation in public health. Health Research Policy and Systems. 2020 Dec;18(1):1-9. | Protocol, theoretical or discussion paper about translation capacity or capability building |
| Augustsson H, Costea VA, Eriksson L, Hasson H, Bäck A, Åhström M, Bergström A. Building implementation capacity in health care and welfare through team training—study protocol of a longitudinal mixed-methods evaluation of the building implementation capacity intervention. Implementation Science Communications. 2021 Dec;2:1-0. | Protocol, theoretical or discussion paper about translation capacity or capability building |
| United States Agency for International Development: Capacity Building for Research Translation | Protocol, theoretical or discussion paper about translation capacity or capability building |
| Gattuso JS, Hinds PS, Beaumont C, Funk AJ, Green J, Max A, Russell P, Windsor K. Transforming a hospital nursing research fellowship into an evidence-based practice fellowship. JONA: The Journal of Nursing Administration. 2007 Dec 1;37(12):539-45. | Program aimed to develop general research skills |
| Joubert L, Hocking A. Academic practitioner partnerships: A model for collaborative practice research in social work. Australian Social Work. 2015 Jul 3;68(3):352-63. | Program aimed to develop general research skills |
| Kajermo KN, Nordström G, Krusebrant A, Lützén K. Nurses' experiences of research utilization within the framework of an educational programme. Journal of Clinical Nursing. 2001 Sep 1;10(5):671-81. | Program aimed to develop general research skills |
| Gerrish K, McDonnell A, Nolan M, Guillaume L, Kirshbaum M, Tod A. The role of advanced practice nurses in knowledge brokering as a means of promoting evidence‐based practice among clinical nurses. Journal of advanced nursing. 2011 Sep;67(9):2004-14. | Program aimed to develop general research skills |
| McWilliam CL. Continuing education at the cutting edge: Promoting transformative knowledge translation. Journal of Continuing Education in the Health Professions. 2007 Mar;27(2):72-9. | Program aimed to develop general research skills |
| Christensen M, Craft J. The nursing professorial unit: translating acute and critical care nursing research. International Practice Development Journal. 2017. | Program aimed to develop general research skills |
| Daudelin DH, Selker HP, Leslie LK. Applying process improvement methods to clinical and translational research: conceptual framework and case examples. Clinical and translational science. 2015 Dec;8(6):779-86. | Program aimed to develop general research skills |
| McArthur A, Munn Z, Lizarondo L, Porritt K, Stephenson M, Stern C, Lockwood C, Moola S, Pearson A, Jordan Z. The ripple effect of evidence implementation: a descriptive evaluation of JBI's Evidence-based Clinical Fellowship Program. JBI Evidence Implementation. 2021 Jun 1;19(2):142-8. | Program implemented in a low- or middle-income country |
| Tripathy JP, Kumar AM, Guillerm N, Berger SD, Bissell K, Reid A, Zachariah R, Ramsay A, Harries AD. Does the Structured Operational Research and Training Initiative (SORT IT) continue to influence health policy and/or practice?. Global Health Action. 2018 Jan 1;11(1):1500762. | Program implemented in a low- or middle-income country |
| Salloum RG, LeLaurin JH, Nakkash R, Akl EA, Parascandola M, Ricciardone MD, Elbejjani M, Kabakian-Khasholian T, Lee JH, El-Jardali F, Shelley D. Developing Capacity in Dissemination and Implementation Research in the Eastern Mediterranean Region: Evaluation of a Training Workshop. Global Implementation Research and Applications. 2022 Nov 14:1-0. | Program implemented in a low- or middle-income country |
| Mittal S. How organizations implement new practices in dynamic context: role of deliberate learning and dynamic capabilities development in health care units. Journal of Knowledge Management. 2019 Jun 26. | Program implemented in a low- or middle-income country |
| Baldwin JA, Williamson HJ, Eaves ER, Levin BL, Burton DL, Massey OT. Broadening measures of success: results of a behavioral health translational research training program. Implementation Science. 2017 Dec;12(1):1-1. | Part of tertiary qualification |
